# Supplementary material for: Exploring stakeholders’ experiences and perceptions regarding barriers to effective surveillance of communicable diseases in a rural district of Pakistan: a qualitative study
Source: BMJ Open. 2022 Nov 11;12(11):e067031. doi: 10.1136/bmjopen-2022-067031 (PMC9659716; doi:10.1136/bmjopen-2022-067031)
Supplement: Author's manuscript [file bmjopen-2022-067031.draft_revisions.pdf]

BMJ Open is committed to open peer review. As part of this commitment we make the peer review history of every article we publish publicly available.

When an article is published we post the peer reviewers' comments and the authors' responses online. We also post the versions of the paper that were used during peer review. These are the versions that the peer review comments apply to.

The versions of the paper that follow are the versions that were submitted during the peer review process. They are not the versions of record or the final published versions. They should not be cited or distributed as the published version of this manuscript.

BMJ Open is an open access journal and the full, final, typeset and author-corrected version of record of the manuscript is available on our site with no access controls, subscription charges or pay-per-view fees (http://bmjopen.bmj.com).

If you have any questions on BMJ Open's open peer review process please email info.bmjopen@bmj.com

BMJ Open

Exploring stakeholders' experiences and perceptions of barriers to effective surveillance of communicable diseases in a rural district of Pakistan – a qualitative study

Journal:	*BMJ Open*
Manuscript ID	bmjopen-2022-067031
Article Type:	Original research
Date Submitted by the Author:	29-Jul-2022
Complete List of Authors:	Naeem, Imran; Aga Khan University Medical College Pakistan, Community Health Sciences Department; Aga Khan University<br>Siddiqi, Sameen; Aga Khan University Medical College Pakistan, Community Health Sciences Department<br>Siddiqui, Amna Rehana; Aga Khan University, Community Health Sciences<br>Hasan, Rumina; The Aga Khan University Hospital Main Campus Karachi; London School of Hygiene and Tropical Medicine Faculty of Public Health and Policy
Keywords:	International health services < HEALTH SERVICES ADMINISTRATION & MANAGEMENT, Organisation of health services < HEALTH SERVICES ADMINISTRATION & MANAGEMENT, Quality in health care < HEALTH SERVICES ADMINISTRATION & MANAGEMENT

SCHOLARONE™
Manuscripts

I, the Submitting Author has the right to grant and does grant on behalf of all authors of the Work (as defined in the below author licence), an exclusive licence and/or a non-exclusive licence for contributions from authors who are: i) UK Crown employees; ii) where BMJ has agreed a CC-BY licence shall apply, and/or iii) in accordance with the terms applicable for US Federal Government officers or employees acting as part of their official duties; on a worldwide, perpetual, irrevocable, royalty-free basis to BMJ Publishing Group Ltd ("BMJ") its licensees and where the relevant Journal is co-owned by BMJ to the co-owners of the Journal, to publish the Work in this journal and any other BMJ products and to exploit all rights, as set out in our licence.

The Submitting Author accepts and understands that any supply made under these terms is made by BMJ to the Submitting Author unless you are acting as an employee on behalf of your employer or a postgraduate student of an affiliated institution which is paying any applicable article publishing charge ("APC") for Open Access articles. Where the Submitting Author wishes to make the Work available on an Open Access basis (and intends to pay the relevant APC), the terms of reuse of such Open Access shall be governed by a Creative Commons licence – details of these licences and which Creative Commons licence will apply to this Work are set out in our licence referred to above.

Other than as permitted in any relevant BMJ Author's Self Archiving Policies, I confirm this Work has not been accepted for publication elsewhere, is not being considered for publication elsewhere and does not duplicate material already published. I confirm all authors consent to publication of this Work and authorise the granting of this licence.

For peer review only

COREQ (COnsolidated criteria for REporting Qualitative research) Checklist

A checklist of items that should be included in reports of qualitative research. You must report the page number in your manuscript where you consider each of the items listed in this checklist. If you have not included this information, either revise your manuscript accordingly before submitting or note N/A.

Topic	Item No.	Guide Questions/Description	Reported on Page No.
**Domain 1: Research team and reflexivity**			
Personal characteristics			
Interviewer/facilitator	1	Which author/s conducted the interview or focus group?	
Credentials	2	What were the researcher's credentials? E.g. PhD, MD	
Occupation	3	What was their occupation at the time of the study?	
Gender	4	Was the researcher male or female?	
Experience and training	5	What experience or training did the researcher have?	
Relationship with participants			
Relationship established	6	Was a relationship established prior to study commencement?	
Participant knowledge of the interviewer	7	What did the participants know about the researcher? e.g. personal goals, reasons for doing the research	
Interviewer characteristics	8	What characteristics were reported about the inter viewer/facilitator? e.g. Bias, assumptions, reasons and interests in the research topic	
**Domain 2: Study design**			
Theoretical framework			
Methodological orientation and Theory	9	What methodological orientation was stated to underpin the study? e.g. grounded theory, discourse analysis, ethnography, phenomenology, content analysis	
Participant selection			
Sampling	10	How were participants selected? e.g. purposive, convenience, consecutive, snowball	
Method of approach	11	How were participants approached? e.g. face-to-face, telephone, mail, email	
Sample size	12	How many participants were in the study?	
Non-participation	13	How many people refused to participate or dropped out? Reasons?	
Setting			
Setting of data collection	14	Where was the data collected? e.g. home, clinic, workplace	
Presence of non-participants	15	Was anyone else present besides the participants and researchers?	
Description of sample	16	What are the important characteristics of the sample? e.g. demographic data, date	
Data collection			
Interview guide	17	Were questions, prompts, guides provided by the authors? Was it pilot tested?	
Repeat interviews	18	Were repeat inter views carried out? If yes, how many?	
Audio/visual recording	19	Did the research use audio or visual recording to collect the data?	
Field notes	20	Were field notes made during and/or after the inter view or focus group?	
Duration	21	What was the duration of the inter views or focus group?	
Data saturation	22	Was data saturation discussed?	
Transcripts returned	23	Were transcripts returned to participants for comment and/or	

For peer review only

Topic	Item No.	Guide Questions/Description	Reported on Page No.
		correction?	
**Domain 3: analysis and findings**			
Data analysis			
Number of data coders	24	How many data coders coded the data?	
Description of the coding tree	25	Did authors provide a description of the coding tree?	
Derivation of themes	26	Were themes identified in advance or derived from the data?	
Software	27	What software, if applicable, was used to manage the data?	
Participant checking	28	Did participants provide feedback on the findings?	
Reporting			
Quotations presented	29	Were participant quotations presented to illustrate the themes/findings? Was each quotation identified? e.g. participant number	
Data and findings consistent	30	Was there consistency between the data presented and the findings?	
Clarity of major themes	31	Were major themes clearly presented in the findings?	
Clarity of minor themes	32	Is there a description of diverse cases or discussion of minor themes?	

Developed from: Tong A, Sainsbury P, Craig J. Consolidated criteria for reporting qualitative research (COREQ): a 32-item checklist for interviews and focus groups. *International Journal for Quality in Health Care*. 2007. Volume 19, Number 6: pp. 349 – 357

Once you have completed this checklist, please save a copy and upload it as part of your submission. DO NOT include this checklist as part of the main manuscript document. It must be uploaded as a separate file.

For peer review only

TITLE:

Exploring stakeholders' experiences and perceptions of barriers to effective surveillance of communicable diseases in a rural district of Pakistan – a qualitative study

Imran Naeem[1*], Sameen Siddiqi[2], Amna Rehana Siddiqui[3], Rumina Hasan[4]

[1]Assistant Professor, Community Health Sciences, Aga Khan University Karachi Pakistan

[2]Professor & Chair, Community Health Sciences, Aga Khan University Karachi Pakistan

[3]Consultant, Community Health Sciences, Aga Khan University Karachi Pakistan

[4]Professor, Pathology & Laboratory Medicine, Aga Khan University Karachi Pakistan

[*]**Corresponding author**

Email: Imran.naeem2009@gmail.com

For peer review only - http://bmjopen.bmj.com/site/about/guidelines.xhtml

ABSTRACT

Objective: To explore the experiences and perceptions of health system stakeholders of a rural district of Sindh, Pakistan regarding the barriers to effective surveillance of communicable diseases.

Design: This exploratory qualitative study comprised of in-depth interviews. An inductive thematic analysis was applied to identify key themes from the data.

Settings: The study was conducted in public sector healthcare facilities and the district health office of the rural district of Thatta, in Sindh province, Pakistan.

Participants: Fifteen healthcare managers and healthcare providers working in the eight public sector primary and secondary healthcare facilities were interviewed using an open-ended in-depth interview guide.

Results: Key themes that emerged from the data were: poor governance and absence of surveillance policy framework; fragmentation in the health system leading to lack of uniform reporting; inadequate (human) resources that weakened the infrastructure for disease surveillance; hospital-based reporting of cases that led to a predominantly passive surveillance system; paper-based surveillance system as the key determinant of delayed reporting; non-utilization of surveillance data for decision making; absence of local laboratory capacity to complement the detection of disease outbreaks and lack of private sector integration in disease surveillance.

Conclusions: Poor governance and lack of policy framework were perceived to be responsible for weak surveillance infrastructure. inadequate resource investment including human resource, paper-based reporting and the absence of local laboratory capacity was considered to result in delayed, poor, and inadequate reporting. The lack of private sector engagement was identified as a major gap.

Keywords: surveillance, communicable diseases, health services research, qualitative

For peer review only

For peer review only - http://bmjopen.bmj.com/site/about/guidelines.xhtml

Strengths and limitations of this study

- The study comprehensively explored the barriers and perceptions to effective disease surveillance by interviewing a diverse group of key stakeholders that were directly engaged in service delivery at the district level
- Adopting a qualitative approach provided a deeper insight into the challenges faced by district health system stakeholders in disease surveillance
- The study was conducted in a predominantly rural district of Pakistan that is already challenged in terms of resource availability. Despite that state of disease surveillance is more or less similar in most districts of Pakistan when generalizing the study findings, these may be interpreted in a similar context.

For peer review only

For peer review only - http://bmjopen.bmj.com/site/about/guidelines.xhtml

BACKGROUND

Infectious diseases continue to pose threat to the health of the public globally. In developing countries, infectious diseases form a significant portion of the disease burden including HIV/AIDS, tuberculosis (TB), malaria, respiratory infections, hepatitis B & C in adults and pneumonia and diarrhoea in children under five years of age [1 2]. In Pakistan, communicable diseases remain a major cause of public health concern with a significant contribution to morbidity and mortality. Conditions like overcrowding, low socio-economic status, poor hygiene and unsafe drinking water and poor awareness of health lead to an environment conducive to disease outbreaks. The Health System of the country is toppled by issues of poor governance and lack of resources resulting in a disease surveillance system that is inept at detecting disease outbreaks [3]. Before the devolution of the health system, Pakistan had two main sources to collect data for health indicators namely: 1) health management information system: this was designed to collect data on selected health indicators from health facilities with established reporting lines from provincial to federal health ministry; 2) data from vertical programs such as national TB control program, malaria control program, HIV/AIDS control program amongst others [4]. Following health system devolution in 2010, administrative powers were devolved to provinces with the district becoming the autonomous unit for defining its health priorities and health planning [5]. In the new, albeit ill-prepared administrative setup district health information system (DHIS) was established to collect disease-related data from the health facility level. In all provinces of the country including the Sindh province, DHIS is the major or only source of information on health indicators of the population presenting to public sector health facilities. However, the DHIS has remained underutilized for communicable disease surveillance. In the last five years, rural districts of Sindh have seen outbreaks of diseases including measles [6] and HIV/AIDS [7] in children pointing out a surveillance system weak at detecting outbreaks. As per the international health regulations, member states of the World Health Organization have the obligation to develop and strengthen disease surveillance with the help of existing health system resources [8]. However, the emergence of recent outbreaks has indicated weaker surveillance of communicable diseases more so in rural areas. Moreover, only those diseases with global priority (such as COVID-19 and Polio) manage to get attention, whereas surveillance for diseases of national priority is often unable to compete with other health

For peer review only - http://bmjopen.bmj.com/site/about/guidelines.xhtml

priorities for policy space and resources. There often is reliance on disease numbers to highlight weak surveillance. But perceptions and experiences of those at the forefront of service delivery are less well studied in Pakistan. Hence, we conducted this study to explore the experiences and perceptions of health system stakeholders of a rural district of Sindh, Pakistan regarding the barriers to effective surveillance of communicable diseases.

METHODS

Study design and setting

This study used a descriptive exploratory design to explore the perceptions and experiences of district health system stakeholders regarding the barriers to an effective surveillance system for communicable diseases in the rural district of Thatta located in the province of Sindh, Pakistan. Thatta is situated approximately 100 kilometres from the provincial capital of Karachi. It is a predominantly rural district with an approximate population of 1 million [9 10]. Health indicators including maternal mortality ratio and neonatal mortality rate are amongst the worst in the country [11 12]. The situation of the healthcare system of Thatta is comparable to any other rural district of Pakistan with inadequate infrastructure and resources. There exist primary and secondary healthcare facilities in the district and there is also a private healthcare system in the district comprising general practitioner clinics and small hospitals. The study was conducted from February 15 to April 30, 2022, in eight public sector primary and secondary healthcare facilities in the district.

Study participant

We used purposive sampling to select study participants. Eligible participants were healthcare managers and healthcare providers working in the eight public sector primary and secondary healthcare facilities. Healthcare managers were those working at the district level and were responsible for the management of health services. Healthcare providers were the doctors responsible for the provision of clinical care at selected healthcare facilities. Participants included both males (n = 12) and females (n = 3). The age range of study participants was 29 – 57 years.

We used the 'saturation principal' to determine the sample size for the study. Upon researchers' observation, with further interviews yielding no added information and

For peer review only

For peer review only - http://bmjopen.bmj.com/site/about/guidelines.xhtml

producing no new or significant findings, data collection was stopped. This resulted in 15 in-depth interviews with healthcare managers and healthcare providers.

Data collection

The data were collected using an open-ended interview guide. The interview guide was used to conduct in-depth interviews for exploring participants' perceptions and experiences with the surveillance system for communicable diseases in the Thatta district. The interview guide was developed by researchers after a thorough literature search on the subject and a review of literature in line with the objective of the research. The interview guide contained open-ended questions regarding perceptions and experiences about the current state of the surveillance system of communicable diseases, challenges faced in terms of resources, infrastructure and financing, and barriers faced in terms of reporting, timeliness, and data quality. The interview guide was piloted before data collection. The interviews were conducted by the first author (IN) who has research experience in health systems and communicable diseases and qualitative research. The first author moderated the interviews along with note-takers. The interviews were conducted in the local language (Sindhi) and were audiotaped. The first author and the note takers held a debriefing session after each interview to reflect on the participants' responses. The interviews took place in health facilities at a time suitable for study participants with each interview lasting about 20 – 35 minutes. Researchers had formal links established with the study respondents due to their four years long work experience with stakeholders of the Thatta district. The study participants were explained the study objective, and any queries raised before data collection were satisfied.

Ethical considerations

Informed written and verbal consent was obtained from all study participants. Ethical approval for this study was obtained from the Ethics Review Committee of the Aga Khan University Karachi, Pakistan (ERC # 2020-5777-15184). Privacy, confidentiality, and anonymity of the respondents were ensured during data collection and reporting of the study findings.

For peer review only

For peer review only - http://bmjopen.bmj.com/site/about/guidelines.xhtml

Researchers' characteristics

In qualitative research, research findings have the potential to be influenced by the study settings and researchers' interest and understanding of the topic. In this study, researchers were not the staff of the health facilities where the study was conducted. However, due to the researchers' four years' work experience in the district, they were considered by the study respondents to be someone familiar with the district health system and its challenges and with an interest in exploring their perceptions and experiences regarding the challenges that the district might be facing in terms of disease surveillance.

Patient and public involvement statement

Patients or the public were not involved in the design, or conduct, or reporting, or dissemination plans of our research

DATA ANALYSIS

We used the inductive method to perform data analysis. The analysis approach used a combination of interviews with study participants facilitated by field observations. The first author transcribed all audio tapes from Sindhi to English and wrote interview notes. Manual content analysis was performed where interview notes were read and re-read to identify similarities and differences of perspectives among study participants. Similar perspectives were grouped and classified into main themes. All the authors and field team members read the themes, and discrepancies were discussed during the interpretation and analysis of data. The themes emerging from the analysis were shared and discussed with study participants for their comments.

RESULTS

Data analysis identified a number of core themes from the experiences and perceptions of healthcare managers and healthcare providers regarding the surveillance system of communicable diseases. Where appropriate, verbatim quotations from the interview transcripts have been used to ensure rigour in the reporting of data. To avoid identifying specific individuals, the participants have been allocated aliases.

The absence of a surveillance policy framework and poor governance leads to an ill-defined disease surveillance system

For peer review only - http://bmjopen.bmj.com/site/about/guidelines.xhtml

All study participants unanimously pointed out the lack of a comprehensive policy framework for disease surveillance. As a result, there is a lack of clarity in reporting disease-related data, coordination between different stakeholders, and meaningful analysis of data and its use for taking action.

"National health policy emphasizes the importance of disease surveillance and having such a system in place; however, it is up to provinces to develop detailed guidelines for disease surveillance and ensure its implementation which is not happening! [Participant 14]."

Provincial disease surveillance and response unit

A digital system named Provincial Disease Surveillance and Response Unit (PDSRU) was developed a few years ago with the support of donor money. The PDSRU had all the communicable diseases of importance listed and the system was expected to be linked up with secondary hospitals in the province for regular data collection. However, interview respondents commented that the PDSRU unit has remained dysfunctional and has not been utilized for the purpose it was built for.

"It is very unfortunate that we got the donor money to establish a surveillance system in the province, but we didn't plan for resources to make the system functional [participant 5]."

District health information system

The DHIS has existed longer than the PDSRU, has the service delivery data collated from primary and secondary health facilities and the frequency of reporting is monthly. The data is collected on paper at the health facility level and a hard copy of the monthly DHIS reporting form is handed over physically by each facility to the district health office. There, a computer operator enters the data into the digital portal of DHIS which then can be viewed on a dashboard.

In the current state, DHIS is the only functional health information system that has up-to-date disease information. However, this information remains underutilized for disease surveillance as there is hardly any review and feedback on the reported data from the district health office or provincial health department. Moreover, since the data is collated and shared at the end of each month, its utility for detecting disease outbreaks is limited.

For peer review only

For peer review only - http://bmjopen.bmj.com/site/about/guidelines.xhtml

"DHIS data can be a very good source of passive surveillance, but we need to increase the reporting frequency from monthly to weekly and have regular reviews of the reported data [Participant 5]."

Communicable Disease Control unit

The Communicable Disease Control (CDC) unit was built at the provincial level with the intent to integrate all the vertical (disease-specific) programs under one roof for planning, resource allocation and intervention. Under the CDC unit, blood-borne diseases like HIV/AIDS, hepatitis C and hepatitis B could be looked after single-handedly when addressing the mode of spread, designing interventions and surveillance. Unfortunately, the unit lacks any infrastructure, dedicated human resources and disease integration plan at the district level.

"Having CDC presents an immediate opportunity for syndromic surveillance; we should not waste this opportunity [Participant 10]."

Fragmentation in the healthcare system is a hindrance to a uniform reporting system

Under the public-private partnership (PPP), the public sector healthcare system in Thatta has been contracted out to various private providers. Under PPP, these providers are responsible for providing health services on a day-to-day basis while the health department provides a budget. However, due to extensive contracting out each level of health facility (i.e., primary, and secondary) is managed by a different private partner. Distinct reporting lines and a lack of data sharing mechanisms between private partners have given rise to fragmentation within the district health system.

"Primary level facilities are supposed to be linked with secondary level facilities for referrals and reporting of disease-related data, however, there are so many partners with each having its reporting line. This has negatively affected the reporting of disease surveillance [participant 10]."

At the district level, surveillance for vaccine-preventable diseases (VPDs) is conducted by the district health office with the district surveillance coordinator as the focal point. Whereas notifiable diseases are reported by the focal persons of the disease-specific programs with a flow of information that is separate from that of VPDs. This vertical nature of surveillance of

For peer review only - http://bmjopen.bmj.com/site/about/guidelines.xhtml

different diseases is inefficient in terms of resource utilization as it creates parallel systems where surveillance activities are carried out in siloes. These inefficiencies in turn create a weaker surveillance system and put the population at higher risk for communicable disease outbreaks.

"Every disease-specific program such as that of tuberculosis, malaria etc reports its data in isolation, how can we have an integrated disease surveillance system in this situation? [Participant 7]."

Inadequate resources translate to poor disease surveillance

In the district health office, there is a district surveillance coordinator who is mandated to look after the surveillance of VPDs in the district. He is solely responsible for coordinating with all eight public sector health facilities of the district, ensuring the timely collection of surveillance data and its reporting. He is also responsible for entering the disease-related information into the district health information system. This not only leads to increased workload but often time leads to delayed data entry and communication of this information to district health stakeholders.

Not only that he lacks a team of dedicated individuals for surveillance, but he also has no provision of transportation for reaching out to health facilities and communities for active surveillance. Most of the time, he has to rely on making telephonic contact with each in charge of health facilities to get disease-related information.

Currently, reporting for VPDs is mandatory and health facilities are required to send weekly reports (even for no cases called 'zero reports') of VPDs to the district surveillance coordinator. However, due to a lack of resources such as transportation and dedicated surveillance staff, the reports from various health facilities in the district often get delayed.

"Expecting one person to lead disease surveillance in the district, in the absence of transportation and adequate human resource is too much to ask [Participant 2]."

For notifiable diseases, the district focal persons of disease-specific programs have the responsibility to report the number of cases of the disease. The primary role of the focal person is program implementation which involves numerous tasks from planning and implementing to monitoring and reporting program activities. In absence of adequate

For peer review only - http://bmjopen.bmj.com/site/about/guidelines.xhtml

human resource, focal persons primarily rely on data reported as part of program implementation. This has limitations namely 1) this is a form of passive surveillance rather than active; 2) reporting of program data takes place at specific intervals whereas surveillance requires continuous monitoring of disease cases and prompt action. These limitations lead to a disease surveillance system that struggles with timely detection, and reporting of disease outbreaks.

The current surveillance system is hospital-based

Study respondents pointed out that since the data collection is passive as it is collected largely from those patients that present to health facilities, hence it is a form of passive surveillance rather than active surveillance.

"We could be missing out on outbreaking at the community level since there is no one going and actively screening the community members [Participant 1]"

Respondent attributed the lack of community-based active surveillance to a lack of resources and mentioned that there is a need to increase resource allocation for strengthening active surveillance.

Paper-based reporting is a key determinant of delayed disease reporting

The system for reporting health-related data from health facility to district health office in Pakistan is paper-based for both vaccine-preventable diseases and notifiable diseases. In this system, the health facility in-charge fills out a zero-reporting form or a case report for a notifiable disease. A physical copy of this report is then sent to the district surveillance officer. This often gets delayed as someone must visit either the health facility or the office of the district surveillance coordinator to deliver the physical copy of the report. To prevent delay, telephonic contact often plays a key role, however, it is not a feasible way to disseminate the information to all the relevant stakeholders.

"We should have a digital system with a dashboard which can show data in real-time. We will avoid delayed reporting forever [participant 2]."

Surveillance data is underutilized for evidence-based decision making

For peer review only - http://bmjopen.bmj.com/site/about/guidelines.xhtml

The majority of study respondents mentioned that data verification is essential to ensure its fidelity. To ensure data quality, monitoring and supervision of field staff and the facility healthcare staff sending data need strengthening.

In the existing system of disease surveillance, there is an emphasis on data collection only. No monitoring and supervision are happening from the district or provincial levels. Other than the circumstances where a suspected or confirmed case of disease under surveillance is reported, there is no action taken to verify the authenticity and quality of routinely reported data.

"Despite that, the data is regularly collected through DHIS and other channels, it is rarely reviewed or utilized for analysis or action [Participant 12]."

Lack of laboratory testing capacity takes a toll on disease surveillance

Among the eight public sector health facilities in the Thatta district, only two have clinical laboratories. However, none of these laboratories is equipped to conduct testing for any of the VPDs or notifiable diseases in the district. Biological samples collected from suspected patients are sent to a regional laboratory that is based in the capital city of the country (laboratory at National Institute of Health, Islamabad) situated at least 1000 kilometres from Thatta district.

The absence of a fully equipped laboratory nearby calls for measures to ensure proper storage and transportation of biological samples. This not only is resource-intensive but adds to the delays in the system staggered by issues of delayed reporting.

"Provincial health department should, at the least, take measures to build a laboratory in the province so that disease surveillance can be made a little more efficient [Participant 6]."

The lack of integration of the private sector in disease surveillance is a major gap

The private health sector is a major stakeholder in service delivery and caters to approximately 70% healthcare needs of the population. Unfortunately, however, the service delivery data of the private health sector is not integrated into the DHIS of the public health sector. In fact, the government is still lagging in taking measures to regulate the private health sector in Sindh province.

For peer review only - http://bmjopen.bmj.com/site/about/guidelines.xhtml

Integration of data from the private sector in the health information system is essential to ensure effective disease surveillance as currently, a significant chunk of the population's disease burden remains to be captured.

"Our disease surveillance will be at a loss from capturing the true disease burden unless it integrates data from the private health sector [Participant 6]."

DISCUSSION

Our study explored multifaceted barriers to effective surveillance of communicable diseases in a rural district of Pakistan. Findings showed that while infrastructure for surveillance at the provincial level is underutilized, structural challenges such as resource deficiencies (including human resource), poor coordination among stakeholders for sharing data, absence of private sector involvement, paper-based reporting, and lack of utilization of data for decision making are some of the key challenges at the district level.

Though it has been over a decade since the devolution of the health system in Pakistan, the pace of provinces leading health planning has remained rather sluggish. Given the increasing disease outbreaks in Sindh and other provinces in recent years i.e., measles, HIV/AIDS and the global COVID pandemic, the need for comprehensive guidelines and laws for disease surveillance has grown stronger. Study participants were concerned about the delay in taking initiatives for the development of surveillance guidelines at the provincial level. This experience is similar to other lower and lower-middle-income countries. Studies from Nigeria and Zambia show that the laws related to public health surveillance existed but were considered to be outdated and/or poorly implemented [13]. Studies from Iran and Palestine concluded that having laws and policies for disease surveillance enable governments to allocate funds for establishing surveillance programs and that health authorities should play a lead role in ensuring their implementation [14 15].

Having a dysfunctional PDSRU for reporting surveillance data is a classic example of relying on donor money instead of concentrating on building local capacity toward a functional information system. Many respondents termed this unfortunate and emphasized investing resources in reviving the PDSRU which was already there. In a study from China inadequacy of resources at the local level was termed as the key factor affecting the effectiveness of disease surveillance. The study reported that the top tier of government invests more in

For peer review only - http://bmjopen.bmj.com/site/about/guidelines.xhtml

building digital information systems, but lower levels don't receive enough planned resources to ensure its implementation [16]. A functional digital information system has been found to speed up the reporting, improve data flow and ensure the availability of up-to-date data for the decision-makers. This eventually leads to early detection of outbreaks and timely action [14]. Having the district surveillance coordinator as the sole person responsible for surveillance activities in the district without any additional resources was considered the major weakness in the surveillance system by study respondents. Literature shows that during an epidemic, there is an increase in the workload requiring more human resource and support for efficient data reporting [17]. The resource deficiencies including field-based staff have been shown to undermine effective disease surveillance [15 18]. Moreover, putting an extra burden of surveillance activities on healthcare providers alongside the service delivery affects their motivation, performance, and quality of reporting [14 16 19].

Despite several advantages of contracting out of health services that the literature notes, the structural challenges largely remain unaddressed [20]. One such structural challenge is poor coordination between stakeholders. Many respondents pointed out that having multiple stakeholders in the same district had been detrimental to disease surveillance due to a lack of coordination and ambiguous reporting lines. The flow of data is independent of the level of health facility defeating the notion of integrated disease surveillance. Similarly, disease-specific programs (malaria, dengue), that have a strong component of environmental control measures, lack integration with the public health engineering department. A study from India attributed the inefficiency in reporting dengue surveillance data to poor integration of health services [18]. Poor coordination between health and other sectors [17] and between various levels of health facilities [14] has been reported to affect not just the data reporting but also may lead to a lack of trust among stakeholders.

The current system for reporting diseases is a form of passive surveillance as it is hospital-based, and data is gathered from patients presenting to health facilities. Literature shows that the majority of people may not opt to show up unless they have serious symptoms [16]. This, in the event of a communicable disease, not only has the potential for the spread of infection but also may cause a delay in detecting an outbreak. Relying on passive surveillance only is often dependent on factors including patients' awareness and health-seeking behaviours and socioeconomic status and hence needs to be supplemented by some form of active surveillance [19].

For peer review only - http://bmjopen.bmj.com/site/about/guidelines.xhtml

Paper-based reporting in our study came out as an important barrier to timely reporting. The need for transitioning from slow, staff reliant and paper-based reporting to the digital mode of reporting is increasingly recognized in literature [14 17] and a study from India has demonstrated improved disease notification and enhanced data reporting due to transitioning to digital media [21].

Except when suspected or a confirmed case of a disease is detected, data sent from health facilities is rarely reviewed at higher levels. Respondents in our study pointed out that there is more emphasis on data collection than its analysis and use. It is evident from the Literature that providing regular feedback to facility staff on the data has been shown to act as a motivating factor and a performance boost [14 18].

Respondents in our study cited the lack of local laboratory capacity in the district as an important gap in detecting disease outbreaks. Studies in literature have demonstrated that in absence of a local laboratory, rapid diagnostics kits (RDTs) may facilitate confirming outbreak until laboratory test results become available [19] thus preventing delays that may incur in the transfer of biological samples. However, despite being cheap, resource investment is still required to ensure their uninterrupted availability [18].

The extent to which a health system can detect disease outbreaks is dependent on its capacity to capture patients' data. In Pakistan, the private health sector caters to the majority of the population's healthcare needs. However, it is largely unregulated, particularly in Sindh province leading to a lack of integration of its patient data with the public sector. Studies show that the poor private sector engagement in disease surveillance is an important issue hampering the surveillance efforts in many countries including India [18 21], Iran [22] and China [19].

LIMITATION

Our study was conducted in a predominantly rural district of Pakistan that is already challenged in terms of resource availability. Hence, when generalizing the study findings, these may be interpreted in a similar context. However, the state of disease surveillance is more or less similar in the country and hence experiences and perceptions given are relatable to several districts of the country.

CONCLUSION

For peer review only - http://bmjopen.bmj.com/site/about/guidelines.xhtml

We concluded that poor governance was perceived to lead to underutilization of existing resources for surveillance whereas lack of a policy framework on surveillance was considered to lead to a poor investment of resources in surveillance infrastructure. The absence of resources and inadequate human resource was identified by respondents as the key determinant of delayed and inadequate reporting leading to delayed detection of disease outbreaks. This was further aggravated by the absence of local laboratory capacity. The existing surveillance system was perceived as largely paper-based, slow, and comprised of hospital-based passive surveillance. The lack of private sector engagement in communicable disease surveillance was perceived as a significant gap.

For peer review only

For peer review only - http://bmjopen.bmj.com/site/about/guidelines.xhtml

Funding

This work was supported by a grant from the World Health Organization grant number 1070479-0

Competing Interests

All authors declare that they have no competing interests

Authors contributions

IN conceived the idea for the manuscript and developed the first draft of the manuscript. All authors subsequently read the manuscript. All authors agreed to the final version of the manuscript.

Acknowledgement

We acknowledge all our participants for their cooperation during the conduct of this study

Checklist for the appropriate reporting statement

We used COnsolidated criteria for REporting Qualitative research (COREQ) checklist while writing the manuscript to ensure rigour in reporting the data for qualitative research

Patient consent for publication

Not applicable

Data availability statement

All the data collected as part of this research study are reported in the manuscript

Word count: 4277

List of abbreviations

TB – Tuberculosis

DHIS – District health information system

PDSRU – Provincial Disease Surveillance and Response Unit

CDC – Communicable Disease Control

PPP – Public-private partnership

VPDs – Vaccine preventable diseases

For peer review only

For peer review only - http://bmjopen.bmj.com/site/about/guidelines.xhtml

References

1. Ward JL, Azzopardi PS, Francis KL, et al. Global, regional, and national mortality among young people aged 10–24 years, 1950–2019: a systematic analysis for the Global Burden of Disease Study 2019. *Lancet* 2021;398(10311):1593-618.
2. Ledesma JR, Ma J, Vongpradith A, et al. Global, regional, and national sex differences in the global burden of tuberculosis by HIV status, 1990–2019: results from the Global Burden of Disease Study 2019. *Lancet Infect Dis* 2022;22(2):222-41.
3. World Health Organization. Communicable disease surveillance and response - Pakistan 2022 [cited 2022 July]. Available from: http://www.emro.who.int/pak/programmes/communicable-disease-a-surveillance-response.html accessed July 2022.
4. Ali M, Horikoshi YJPJMR. Situation analysis of health management information system in Pakistan. *Pakistan Journal Medical Research* 2002;41(2):64-9.
5. Zaidi SA, Bigdeli M, Langlois EV, et al. Health systems changes after decentralisation: progress, challenges and dynamics in Pakistan. *BMJ Glob Health* 2019;4(1):e001013.
6. Ahmad Z, Zahid HJJotPMA. Threat of a Measles outbreak amidst COVID 19 surge in Sindh, Pakistan-Letter to the editor. *J Pak Med Assoc* 2021;71(10):2488-88.
7. Siddiqui AR, Nathwani AA, Abidi SH, et al. Investigation of an extensive outbreak of HIV infection among children in Sindh, Pakistan: protocol for a matched case–control study. *BMJ Open* 2020;10(3):e036723.
8. World Health Organization. Inernational Health Regulations 2005 2022 [cited 2022 July]. Available from: http://apps.who.int/iris/bitstream/handle/10665/246107/9789241580496-eng.pdf;jsessionid=95E436B7F04EA4A8635945E4C684A14C?sequence=1 accessed July 2022.
9. Abbasi IN, Siddiqi S. Overcoming the challenges of community-engaged emergency referrals in a rural district of Pakistan. *J Pak Med Assoc* 2021
10. Pakistan Bureau of Statistics. Thatta district 2017 [cited 2022 July]. Available from: https://www.pbs.gov.pk/census-2017-district-wise/results/091 accessed July 2022.
11. Pasha O, Saleem S, Ali S, et al. Maternal and 347 newborn outcomes in Pakistan compared to other low and middle income 348 countries in the Global Network's Maternal Newborn Health Registry: an 349 active, community-based, pregnancy surveillance mechanism. *Reprod Health* 2015;350:12.
12. National Institute of Population Studies Islamabad Pakistan. Pakistan Demographic and Health Survey 2017-18 [cited 2022 July]. Available from: https://dhsprogram.com/pubs/pdf/FR354/FR354.pdf accessed July 2022.
13. Makinde OA, Odimegwu COJPAMJ. A qualitative inquiry on the status and adequacy of legal instruments establishing infectious disease surveillance in Nigeria. *Pan Afr Med J* 2018;31(1)
14. Dehcheshmeh NF, Arab M, Foroushani AR, et al. Survey of communicable diseases surveillance system in hospitals of Iran: A Qualitative approach. *Glob J Health Sci* 2016;8(9):44.
15. Abuzerr S, Zinszer K, Assan AJSom. Implementation challenges of an integrated One Health surveillance system in humanitarian settings: A qualitative study in Palestine. *SAGE Open Med* 2021;9:20503121211043038.
16. Yan W, Zhou Y, Wei S, et al. The difficulties of early detection for infectious disease outbreak in China: a qualitative investigation. *Journal of Nanjing Medical University* 2008;22(1):66-70.
17. Angelo M, Ramalho WM, Gurgel H, et al. Dengue surveillance system in Brazil: A qualitative study in the federal district. *Int J Environ Res Public Health* 2020;17(6):2062.
18. Pilot E, Murthy G, Nittas VJGPH. Understanding India's urban dengue surveillance: A qualitative policy analysis of Hyderabad district. *Glob Public Health* 2020;15(11):1702-17.
19. Lu G, Liu Y, Beiersmann C, et al. Challenges in and lessons learned during the implementation of the 1-3-7 malaria surveillance and response strategy in China: a qualitative study. *Infect Dis Poverty* 2016;5(1):1-11.

For peer review only - http://bmjopen.bmj.com/site/about/guidelines.xhtml

20. Lagarde M, Palmer NJCDoSR. The impact of contracting out on health outcomes and use of health services in low and middle-income countries. *Cochrane Database Syst Rev* 2009(4)
21. Prajitha KC, Rahul A, Chintha S, et al. Strategies and challenges in Kerala's response to the initial phase of COVID-19 pandemic: a qualitative descriptive study. *BMJ Open* 2021;11(7):e051410.
22. Asadi H, Gouya M-M, Nabavi M, et al. The communicable diseases surveillance system in Iran: challenges and opportunities. *Arch Iran Med* 2019

For peer review only

For peer review only - http://bmjopen.bmj.com/site/about/guidelines.xhtml

BMJ Open

Exploring stakeholders' experiences and perceptions regarding barriers to effective surveillance of communicable diseases in a rural district of Pakistan – a qualitative study

Journal:	*BMJ Open*
Manuscript ID	bmjopen-2022-067031.R1
Article Type:	Original research
Date Submitted by the Author:	03-Oct-2022
Complete List of Authors:	Naeem, Imran; Aga Khan University Medical College Pakistan, Community Health Sciences Department; Aga Khan University<br>Siddiqi, Sameen; Aga Khan University Medical College Pakistan, Community Health Sciences Department<br>Siddiqui, Amna Rehana; Aga Khan University, Community Health Sciences<br>Hasan, Rumina; The Aga Khan University Hospital Main Campus Karachi; London School of Hygiene and Tropical Medicine Faculty of Public Health and Policy
**Primary Subject Heading**:	Health services research
Secondary Subject Heading:	Health services research
Keywords:	International health services < HEALTH SERVICES ADMINISTRATION & MANAGEMENT, Organisation of health services < HEALTH SERVICES ADMINISTRATION & MANAGEMENT, Quality in health care < HEALTH SERVICES ADMINISTRATION & MANAGEMENT, Public health < INFECTIOUS DISEASES

SCHOLARONE™
Manuscripts

I, the Submitting Author has the right to grant and does grant on behalf of all authors of the Work (as defined in the below author licence), an exclusive licence and/or a non-exclusive licence for contributions from authors who are: i) UK Crown employees; ii) where BMJ has agreed a CC-BY licence shall apply, and/or iii) in accordance with the terms applicable for US Federal Government officers or employees acting as part of their official duties; on a worldwide, perpetual, irrevocable, royalty-free basis to BMJ Publishing Group Ltd ("BMJ") its licensees and where the relevant Journal is co-owned by BMJ to the co-owners of the Journal, to publish the Work in this journal and any other BMJ products and to exploit all rights, as set out in our licence.

The Submitting Author accepts and understands that any supply made under these terms is made by BMJ to the Submitting Author unless you are acting as an employee on behalf of your employer or a postgraduate student of an affiliated institution which is paying any applicable article publishing charge ("APC") for Open Access articles. Where the Submitting Author wishes to make the Work available on an Open Access basis (and intends to pay the relevant APC), the terms of reuse of such Open Access shall be governed by a Creative Commons licence – details of these licences and which Creative Commons licence will apply to this Work are set out in our licence referred to above.

Other than as permitted in any relevant BMJ Author's Self Archiving Policies, I confirm this Work has not been accepted for publication elsewhere, is not being considered for publication elsewhere and does not duplicate material already published. I confirm all authors consent to publication of this Work and authorise the granting of this licence.

For peer review only

TITLE:

Exploring stakeholders' experiences and perceptions regarding barriers to effective surveillance of communicable diseases in a rural district of Pakistan – a qualitative study

Imran Naeem[1*], Sameen Siddiqi[2], Amna Rehana Siddiqui[3], Rumina Hasan[4]

[1]Assistant Professor, Community Health Sciences, Aga Khan University Karachi Pakistan

[2]Professor & Chair, Community Health Sciences, Aga Khan University Karachi Pakistan

[3]Consultant, Community Health Sciences, Aga Khan University Karachi Pakistan

[4]Professor, Pathology & Laboratory Medicine, Aga Khan University Karachi Pakistan

[*]**Corresponding author**

Email: Imran.naeem@aku.edu

For peer review only

ABSTRACT

Objective: To explore the experiences and perceptions of health system stakeholders of a rural district of Sindh, Pakistan regarding the barriers to effective surveillance of communicable diseases.

Design: This qualitative descriptive exploratory design comprised in-depth interviews. Both inductive and deductive thematic analysis was applied to identify key themes from the data.

Settings: The study was conducted in public sector healthcare facilities and the district health office of the rural district of Thatta, in Sindh province, Pakistan.

Participants: Fifteen healthcare managers and healthcare providers working in the eight public sector primary and secondary healthcare facilities were interviewed using an open-ended in-depth interview guide.

Results: Key themes that emerged from the data were: poor governance and absence of surveillance policy framework; fragmentation in the health system leading to lack of uniform reporting; inadequate (human) resources that weakened the infrastructure for disease surveillance; hospital-based reporting of cases that led to a predominantly passive surveillance system; paper-based surveillance system as the key determinant of delayed reporting; non-utilization of surveillance data for decision making; absence of local laboratory capacity to complement the detection of disease outbreaks and lack of private sector integration in disease surveillance.

Conclusions: Poor governance and lack of policy framework were perceived to be responsible for weak surveillance infrastructure. Resource deficiencies including inadequate human resource, paper-based reporting and the absence of local laboratory capacity were considered to result in delayed, poor quality, and incomplete reporting. The lack of private sector engagement was identified as a major gap.

Keywords: surveillance, communicable diseases, health services research, qualitative, rural, Pakistan

For peer review only

Strengths and limitations of this study

- The study has explored experiences and perceptions regarding barriers to effective surveillance of communicable diseases in-depth by involving representatives from various levels of the healthcare system including from the public health sector and those working under public-private partnership
- The inclusion of both healthcare managers and healthcare providers in the study provided deeper insights into barriers at both the stewardship level and the operational level
- The study is amongst few in Pakistan to adopt a qualitative research approach for exploring barriers to infectious disease surveillance as perceived and experienced by health system stakeholders

For peer review only

For peer review only - http://bmjopen.bmj.com/site/about/guidelines.xhtml

BACKGROUND

Infectious diseases continue to pose threat to the health of the public globally. In developing countries, infectious diseases form a significant portion of the disease burden including HIV/AIDS, tuberculosis (TB), malaria, respiratory infections, hepatitis B & C in adults and pneumonia and diarrhoea in children under five years of age [1 2]. In Pakistan, communicable diseases remain a major cause of public health concern with a significant contribution to morbidity and mortality. Conditions like overcrowding, low socio-economic status, poor hygiene and unsafe drinking water and poor awareness of health lead to an environment conducive to disease outbreaks. The Health System of the country is overwhelmed by issues of poor governance and lack of resources resulting in a surveillance system that is ill-equipped at detecting outbreaks of infectious disease [3]. Before the devolution of the health system, Pakistan had two main sources to collect data for health indicators namely: 1) health management information system: this was designed to collect data on selected health indicators from health facilities with established reporting lines from provincial to federal health ministry; 2) data from vertical programs such as national TB control program, malaria control program, HIV/AIDS control program amongst others that also reported to federal health ministry [4]. Following health system devolution in 2010, administrative powers were devolved to provinces with the district becoming the autonomous unit for defining its health priorities and health planning [5]. In the new, albeit ill-prepared administrative setup district health information system (DHIS) was established to collect disease-related data from the health facility level [6]. In all provinces of the country including the Sindh province, DHIS is the only source of information on health indicators of the population based on service delivery data from public sector health facilities. However, the DHIS has remained underutilized for communicable disease surveillance. In the last five years, rural districts of Sindh have witnessed outbreaks of diseases such as measles [7] and HIV/AIDS [8] while the surveillance system was unable to predict these outbreaks. As per the international health regulations, member states of the World Health Organization have the obligation to develop and strengthen disease surveillance with the help of existing health system resources [9]. However, the emergence of recent outbreaks has indicated weaker surveillance of communicable diseases more so in rural areas of Pakistan. Moreover, only diseases with global priority (such as COVID-19 and Polio) have managed to get attention, whereas

For peer review only - http://bmjopen.bmj.com/site/about/guidelines.xhtml

surveillance for diseases of national priority has often struggled to compete for policy space and resources. The scientific literature on infectious disease surveillance in Pakistan is either quantitative in nature or mostly has discussed the implementation of models like the disease early warning system (DEWS) for surveillance [10-12]. However, there is a dearth of literature regarding the challenges that healthcare managers and providers face when implementing disease surveillance programs and perceptions and experiences of these healthcare professionals regarding barriers to effective surveillance of infectious diseases are less well studied in Pakistan. Such information will not only give an in-depth insight into the challenges faced in infectious disease surveillance but also inform the policy makers with recommendations for addressing those challenges. Hence, we conducted this study to explore the experiences and perceptions of health system stakeholders of a rural district of Sindh, Pakistan regarding the barriers to effective surveillance of communicable diseases.

METHODS

Study design and setting

This study used a qualitative descriptive exploratory design to explore the perceptions and experiences of district health system stakeholders regarding the barriers to an effective surveillance system for communicable diseases in the rural district of Thatta located in the province of Sindh, Pakistan. Thatta is situated approximately 100 kilometres from the provincial capital of Karachi. It is a predominantly rural district with an approximate population of 1 million [13 14]. Health indicators including maternal mortality ratio and neonatal mortality rate are amongst the worst in the country [15 16]. The situation of the healthcare system of Thatta is comparable to any other rural district of Pakistan with inadequate infrastructure and resources. There exist primary and secondary healthcare facilities in the district and there is also a private healthcare system in the district comprising general practitioner clinics and small hospitals. The study was conducted from February 15 to April 30, 2022, in eight public sector primary and secondary healthcare facilities in the district.

Study participants

We used purposive sampling to select study participants. Eligible participants were healthcare managers and healthcare providers working in the eight public sector primary

For peer review only - http://bmjopen.bmj.com/site/about/guidelines.xhtml

and secondary healthcare facilities. Healthcare managers were those working at the district level and were responsible for the management of health services. Healthcare providers were the doctors responsible for the provision of clinical care at selected healthcare facilities. Participants included both males (n = 12) and females (n = 3). The age range of study participants was 29 – 57 years. The experience of study participants with the district health system ranged from 5 – 30 years.

We used the 'saturation principle' to determine the sample size for the study. Upon researchers' observation, with further interviews yielding no new or significant findings, data collection was stopped. This resulted in 15 in-depth interviews with healthcare managers and healthcare providers.

Data collection

The data were collected using an open-ended interview guide. The guide comprised questions and probes regarding perceptions and experiences about the current state of communicable disease surveillance and challenges in infrastructure and financing for surveillance, barriers in data reporting, timeliness of reporting, and quality of reported data. The guide was developed following a thorough literature review and researchers' own experience and expertise on the subject. The guide was piloted before data collection. Based on the pretest results, researchers gained new insights and revised the interview guide by adding further questions and probes. The interviews were conducted by the first author (IN) having research experience in health systems, communicable diseases, and qualitative research. The first author moderated the interviews along with note-takers. The interviews were conducted in the local language (Sindhi) and were audiotaped. A debriefing session was held after each interview to reflect on the participants' responses. The interviews took place in health facilities at a time suitable for study participants with each interview lasting about 40 – 50 minutes.

Ethical considerations

Informed written and verbal consent was obtained from all study participants and queries raised were satisfied. Ethical approval for this study was obtained from the Ethics Review Committee of the Aga Khan University Karachi, Pakistan (ERC # 2020-5777-15184). Privacy,

For peer review only - http://bmjopen.bmj.com/site/about/guidelines.xhtml

confidentiality, and anonymity of the respondents were ensured during data collection and reporting of the study findings.

Researchers' reflexivity

In qualitative research, research findings are liable to be influenced by researchers' interest and understanding of the topic. In this study, researchers were not the staff of the health facilities where the study was conducted. However, due to the research team's four years' work experience in the district, they were not considered outsiders by the study respondents but rather someone familiar with the district health system and interested in exploring their views. Moreover, researchers used reflexive notes during data collection that fed into the interpretation of study findings to minimize researcher bias.

Researchers had knowledge of the health system and infectious diseases research which influenced the development of the interview guide, however, while pre-testing the guide researchers gained new knowledge which informed the revision of the interview guide.

Patient and public involvement statement

Patients or the public were not involved in the design, or conduct, or reporting, or dissemination plans of our research

DATA ANALYSIS

We used both inductive and deductive methods to perform data analysis. The analysis approach used a combination of interviews with study participants facilitated by field observations. Researchers' background knowledge of health systems and infectious disease research guided the process of data collection and analysis using the deductive method. However, during the analysis new themes emerged that were analysed using the inductive method. All audio tapes were transcribed from Sindhi to English and interview notes were written. Manual content analysis was performed where interview notes and notes from audio transcripts were read and re-read to identify patterns in data. Manual codes were assigned to identified patterns in data which were subsequently grouped and classified into main themes. All the authors and field team members read the themes, and discrepancies were discussed during the interpretation and analysis of data. The themes emerging from the analysis were shared and discussed with study participants for their comments.

For peer review only - http://bmjopen.bmj.com/site/about/guidelines.xhtml

179 **RESULTS**

180 Data analysis identified a number of core themes from in-depth interviews. Where
181 appropriate, direct quotes from the interviews have been used to ensure rigour in the data
182 reporting. To avoid identifying specific individuals, the participants have been allocated
183 aliases. Table 1 summarizes the themes and codes that emerged from the data.

Table 1. Themes and codes emerging from data

Themes	Codes
The absence of a surveillance policy framework and poor governance leads to an ill-defined disease surveillance system	• Lack of provincial policy on infectious disease surveillance<br>• Lack of laws<br>• Lack of surveillance standards<br>• Lack of resource planning leading to dysfunctional digital information systems<br>• Lack of planning to ensure integrated surveillance of infectious diseases
Fragmentation in the healthcare system is a hindrance to a uniform reporting system	• Poor coordination between health system stakeholders<br>• Lack of integration between different levels of health facilities<br>• Lack of defined reporting lines for surveillance data<br>• Different organization managing various levels of health facilities in the district
Inadequate resources translate to poor disease surveillance	• Inadequate provision of facilities and equipment<br>• Lack of dedicated human resource for surveillance<br>• Lack of financial support<br>• Lack of dedicated line item for surveillance in provincial/district budget
In the current system surveillance is predominantly passive	• Hospital based surveillance<br>• Surveillance data collected from patients presenting to hospitals<br>• Lack of human resource to conduct surveillance in communities<br>• Low level of surveillance activities outside hospitals

For peer review only - http://bmjopen.bmj.com/site/about/guidelines.xhtml

Paper-based reporting is a key determinant of delayed disease reporting	• Surveillance data compiled on hard copies<br>• Hard copies are delivered from health facilities to district health office<br>• No dedicated human resource to transfer health facility reports to district health office
Surveillance data is underutilized for evidence-based decision making	• Monthly report submission by health facilities to district health office is mandatory<br>• No feedback provided from district health office to facilities on submitted reports<br>• Submitted reports are not reviewed for data errors<br>• No one from district or provincial makes monitoring visits to check fidelity of reported data
Lack of laboratory testing capacity takes a toll on disease surveillance	• There is no laboratory capable of conducting tests for diseases under surveillance<br>• Samples are sent to regional laboratory for testing
The lack of integration of the private sector in disease surveillance is a major gap	• At provincial or district level, no measures are taken to bring surveillance data from private health sector into mainstream<br>• Private health sector does not report surveillance data to district health office or provincial health department except in case of COVID

The absence of a surveillance policy framework and poor governance leads to an ill-defined disease surveillance system

All study participants unanimously pointed out the lack of a comprehensive policy framework for disease surveillance. A respondent mentioned that despite that following devolution in the health system where the province has the autonomy of decision making, no initiative regarding disease surveillance policy has been taken. As a result, there is a lack

For peer review only

of clarity in reporting disease-related data, coordination between different stakeholders, and meaningful analysis of data and its use for taking action.

"National health policy emphasizes the importance of disease surveillance and having such a system in place; however, it is up to provinces to develop detailed guidelines for disease surveillance and ensure its implementation which is not happening! [Participant 14]."

Provincial disease surveillance and response unit

One of the study respondents mentioned that a digital information system named Provincial Disease Surveillance and Response Unit (PDSRU) was developed in 2016 with the support of donor money. The PDSRU had all the communicable diseases of importance listed and the system was expected to be linked up with secondary hospitals in the province for regular data collection. However, the PDSRU unit has remained dysfunctional and has not been utilized for the purpose it was built for.

"It is very unfortunate that we got the donor money to establish a surveillance system in the province, but we didn't plan for resources to make the system functional [participant 5]."

District health information system

One of the respondents said that the DHIS was developed and implemented in 2010 [6]. It was meant to collate service delivery data from primary and secondary health facilities with a monthly reporting frequency. The data is collected on paper at the health facility level and a hard copy of the monthly DHIS reporting form is handed over physically by each facility to the district health office. There, a computer operator enters the data into the digital portal of DHIS which then can be viewed on a dashboard.

In the current state, DHIS is the only functional health information system that has up-to-date disease information. But a study respondent pointed out that this information remains underutilized for disease surveillance as there is hardly any review and feedback on the reported data from higher levels (i.e., district health office or provincial health department). Moreover, since the data is collated and shared at the end of each month, its utility for detecting disease outbreaks is limited.

For peer review only - http://bmjopen.bmj.com/site/about/guidelines.xhtml

For peer review only

"DHIS data can be a very good source of passive surveillance, but we need to increase the reporting frequency from monthly to weekly and have regular reviews of the reported data [Participant 5]."

Communicable Disease Control unit

A study respondent mentioned that for infectious diseases, a Communicable Disease Control (CDC) unit was established at the provincial level in 2015. The intent was to integrate all the vertical (disease-specific) programs under one roof for planning, resource allocation and public health interventions. Under the CDC unit, blood-borne diseases like HIV/AIDS, hepatitis C and hepatitis B could be looked after single-handedly when addressing the mode of spread, designing interventions and surveillance. Unfortunately, at the district level, the CDC unit lacks infrastructure (e.g., dedicated building and office space, computers, and internet), dedicated human resources (district focal CDC person) and a plan ensuring how the integration of various diseases can take place.

"Having CDC presents an immediate opportunity for syndromic surveillance; we should not waste this opportunity [Participant 10]."

Fragmentation in the healthcare system is a hindrance to a uniform reporting system

Under the public-private partnership (PPP), the public sector healthcare system in Thatta has been contracted out to various private providers. Under PPP, these providers are responsible for providing health services on a day-to-day basis while the health department provides a budget[17].

One of the study respondents mentioned that in the Thatta district extensive contracting out of health services has been done and that each level of health facility (i.e., primary, and secondary) is managed by a different private partner. Distinct reporting lines and a lack of data-sharing mechanisms between private partners have given rise to fragmentation within the district health system.

"Primary level facilities are supposed to be linked with secondary level facilities for referrals and reporting of disease-related data, however, there are so many partners with each having its reporting line. This has negatively affected the reporting of disease surveillance [participant 10]."

For peer review only - http://bmjopen.bmj.com/site/about/guidelines.xhtml

A healthcare manager pointed out that in the Thatta district, surveillance for vaccine-preventable diseases (VPDs) is conducted by the district health office with the district surveillance coordinator as the focal point. Whereas notifiable diseases are reported by the focal persons of the disease-specific programs with a flow of information that is separate from that of VPDs. This vertical nature of surveillance of different diseases is inefficient in terms of resource utilization as it creates parallel systems where surveillance activities are carried out in siloes. These inefficiencies in turn create a weaker surveillance system and put the population at higher risk for communicable disease outbreaks.

"Every disease-specific program such as that of tuberculosis, malaria etc reports its data in isolation, how can we have an integrated disease surveillance system in this situation? [Participant 7]."

Inadequate resources translate to poor disease surveillance

A healthcare manager highlighted the dearth of human resource for surveillance in the district. He mentioned that in the district health office, there is only one person (district surveillance coordinator) who is tasked to coordinate for surveillance of vaccine-preventable diseases (VPDs), data reporting and data entry into DHIS for all eight public sector health facilities of the district. The increased workload results in delayed data entry and delay in data transfer to district health stakeholders. Not only that he lacks a team of dedicated individuals for surveillance, but he also has no provision of transportation for reaching out to health facilities and communities for active surveillance. Most of the time, he has to rely on making telephonic contact with each in charge of health facilities to get disease-related information.

All the study respondents mentioned that reporting for VPDs is mandatory and health facilities are required to send weekly reports (even for no cases called 'zero reports') of VPDs to the district surveillance coordinator. But due to a lack of resources e.g., transportation and dedicated surveillance staff, the reports from various health facilities in the district often get delayed.

"Expecting one person to lead disease surveillance in the district, in the absence of transportation and adequate human resource is too much to ask [Participant 2]."

For peer review only - http://bmjopen.bmj.com/site/about/guidelines.xhtml

A district healthcare manager pointed out that for notifiable diseases, the district focal persons of disease-specific programs have the responsibility of reporting data. The primary role of the focal person is program implementation which involves numerous tasks from planning and implementing to monitoring and reporting program activities. In absence of adequate human resource, focal persons primarily rely on data reported as part of program implementation. This has limitations namely 1) this is a form of passive surveillance rather than active; 2) reporting of program data takes place at specific intervals whereas surveillance requires continuous monitoring of disease cases and prompt action. Many study respondents believed that these limitations lead to a disease surveillance system that struggles with timely detection, and reporting of disease outbreaks.

In the current system, surveillance is predominantly passive

Study respondents pointed out that since the data collection is passive as it is collected largely from those patients that present to health facilities, hence it is a form of passive surveillance rather than active surveillance.

"We could be missing out on outbreaking at the community level since there is no one going and actively screening the community members [Participant 1]"

Respondents attributed the lack of community-based active surveillance to the lack of resources and mentioned that there is a need to increase resource allocation for strengthening active surveillance.

Paper-based reporting is a key determinant of delayed disease reporting

Several study respondents attributed the delayed reporting of surveillance data to paper-based reporting. The system for reporting health-related data from health facilities to the district health offices in Pakistan is paper-based for both VPDs and notifiable diseases. The health facility in charge fills out a zero-reporting form (for VPDs) or a case report (for a notifiable disease). A physical copy of this report is then sent to the district surveillance officer. This often gets delayed as someone must visit the health facility or district surveillance coordinator to deliver the physical copy of the report. To prevent delay,

For peer review only

telephonic contact often plays a key role, however, it is not a feasible way to disseminate the information to all the relevant stakeholders.

"We should have a digital system with a dashboard which can show data in real time. We will avoid delayed reporting forever [participant 2]."

Surveillance data is underutilized for evidence-based decision making

Most study respondents mentioned that data verification is essential to ensure its fidelity. To ensure data quality, monitoring and supervision of field staff and the facility healthcare staff sending data need strengthening. But all respondents agreed that in the existing system of disease surveillance, there is an emphasis on data collection only. No monitoring and supervision are happening from the district or provincial levels. Other than the circumstances where a suspected or confirmed case of disease under surveillance is reported, there is no action taken to verify the authenticity and quality of routinely reported data.

"Despite that, the data is regularly collected through DHIS and other channels, it is rarely reviewed or utilized for analysis or action [Participant 12]."

Lack of laboratory testing capacity takes a toll on disease surveillance

All the study respondents showed their concern regarding poor laboratory capacity for surveillance in the district. Among the eight public sector health facilities in the Thatta district, only two have clinical laboratories. However, none of these laboratories is equipped to conduct testing for any of the VPDs or notifiable diseases in the district. Biological samples collected from suspected patients are sent to a regional laboratory that is based in the capital city of the country (laboratory at National Institute of Health, Islamabad) situated at least 1000 kilometres from Thatta district.

A healthcare manager pointed out that the absence of a fully equipped laboratory nearby calls for measures to ensure proper storage and transportation of biological samples. This not only is resource-intensive but adds to the delays in the system staggered by issues of delayed reporting.

"Provincial health department should, at the least, take measures to build a laboratory in the province so that disease surveillance can be made a little more efficient [Participant 6]."

For peer review only - http://bmjopen.bmj.com/site/about/guidelines.xhtml

For peer review only

The lack of integration of the private sector in disease surveillance is a major gap

All the study respondents considered the absence of private sector integration as an important gap. The private health sector is a major stakeholder in service delivery and caters to approximately 70% healthcare needs of the population. Unfortunately, however, the service delivery data of the private health sector is not integrated into the DHIS of the public health sector. In fact, the government is still lagging in taking measures to regulate the private health sector in Sindh province.

One of the respondents said that integration of data from the private sector in the health information system is essential to ensure effective disease surveillance as currently, a significant chunk of the population's disease burden remains to be captured.

"Our disease surveillance will be at a loss from capturing the true disease burden unless it integrates data from the private health sector [Participant 6]."

DISCUSSION

Our study explored multifaceted barriers to effective surveillance of communicable diseases in a rural district of Pakistan. Our study showed that the lack of policy guidelines at the provincial level was fundamental to ineffective disease surveillance and poor data reporting. Poor stakeholders' coordination led to a lack of sharing of surveillance data hampering the surveillance efforts. Due to poor resource planning, the digital information systems i.e., PDSRU and DHIS built using donor resources were underutilized. Having the district surveillance coordinator as the sole person responsible for surveillance activities in the district, in absence of additional resources, was considered a major resource gap by study respondents. Most study respondents were concerned about the predominantly passive nature of existing district surveillance. Paper-based reporting together with inadequate human resource was considered an important cause of delayed reporting in surveillance. The lack of laboratory testing capacity in the district was another determinant for delayed reporting. There was an increasing emphasis on collecting data than using it for predicting outbreaks or taking measures to control these. The absence of inclusion of infectious disease surveillance data of private sector data in the district surveillance was identified as another major gap by study respondents.

For peer review only - http://bmjopen.bmj.com/site/about/guidelines.xhtml

Despite that a decade has elapsed since the health system in Pakistan was devolved, the pace of provinces taking charge of health planning and resource generation has remained rather sluggish. A study respondent mentioned that despite the increasing infectious disease outbreaks in Sindh and other provinces in recent years i.e., measles, HIV/AIDS and the global COVID pandemic, initiatives regarding comprehensive guidelines and laws for disease surveillance are still lacking. Studies from Nigeria and Zambia show that the laws related to public health surveillance existed but were considered to be outdated and/or poorly implemented [18]. Studies from Iran and Palestine concluded that having laws and policies for disease surveillance enable governments to allocate funds for establishing surveillance programs and that health authorities should play a lead role in ensuring their implementation [10 19].

Having a dysfunctional PDSRU for reporting surveillance data is a classic example of relying on donor money instead of concentrating on building local capacity for a functional health information system. Many respondents termed this unfortunate and emphasized investing resources in reviving the PDSRU. In a study from China inadequacy of resources at the local level was identified as an important determinant of a functional digital information system for disease surveillance. The study reported that the top tier of government invests more in building digital information systems, but lower levels don't receive enough planned resources to ensure its implementation [20]. A functional digital information system has been found to speed up the reporting, improve data flow and ensure the availability of up-to-date data for the decision-makers. This eventually leads to early detection of and timely action against outbreaks [10].

Respondents in our study found the lack of adequate human resource for surveillance at the district level as concerning. Literature shows that for surveillance to be effective, adequate human resource is essential to undertake field-based surveillance and for efficient data reporting [21]. The resource deficiencies including field-based staff have been shown to undermine effective disease surveillance [19 22]. Moreover, putting the burden of surveillance activities on healthcare staff engaged in service delivery negatively affects their motivation, performance, and consequently the quality of reporting [10 20 23].

Despite several advantages of contracting out of health services that the literature notes, the structural challenges largely remain unaddressed [24]. One such challenge is poor coordination between stakeholders. Many respondents pointed out that having multiple

For peer review only - http://bmjopen.bmj.com/site/about/guidelines.xhtml

stakeholders in the same district had been detrimental to disease surveillance due to a lack of coordination and ambiguous reporting lines. The flow of data is independent of the level of health facility defeating the notion of integrated disease surveillance. Poor coordination between stakeholders [21 22] and between different levels of health facilities [10] have been reported to adversely affect the data reporting for disease surveillance.

Many study respondents pointed out the need to strengthen active surveillance in the district as in the present system, the surveillance was largely hospital-based where the data was being gathered from patients presenting to health facilities. Literature shows that the majority of people may opt not to show up for health seeking unless they develop serious symptoms [20]. This, in the event of a communicable disease, not only has the potential for the infection to spread but also leads to a delay in detecting an outbreak. Relying on passive surveillance only is often dependent on factors including patients' awareness, health-seeking behaviour and socioeconomic status and hence needs to be supplemented by some form of active surveillance [23].

Paper-based reporting in our study came out as an important barrier to timely reporting. The need for transitioning from slow, staff-reliant and paper-based reporting to the digital mode of reporting is increasingly recognized in literature [10 21]. A study from India has demonstrated improved disease notification and enhanced data reporting due to transitioning to digital media [25].

Except when suspected or a confirmed case of a disease is detected, data sent from health facilities is rarely reviewed at higher levels. Respondents in our study pointed out that there is more emphasis on data collection than its analysis and use. It is evident from the literature that providing regular feedback to facility staff on the data has been shown to act as a motivating factor and a performance boost [10 22].

Study respondents emphasized the need for having local laboratory capacity to ensure timely detection of disease outbreaks. Studies in literature have demonstrated that in absence of a local laboratory, rapid diagnostics kits (RDTs) may facilitate confirming outbreak until laboratory test results become available [23] thus preventing delays that may incur in the transfer of biological samples [22].

The extent to which a health system can detect disease outbreaks is dependent on its capacity to capture patients' data. In Pakistan, the private health sector caters to most of

For peer review only - http://bmjopen.bmj.com/site/about/guidelines.xhtml

the population's healthcare needs. However, it is largely unregulated in Sindh province and in Pakistan in general, leading to a lack of integration of its patient data with the public sector. Studies show that the poor private sector engagement in disease surveillance is an important issue hampering the surveillance efforts in many countries including India [22 25], Iran [26] and China [23].

LIMITATIONS OF THE STUDY

Our study was conducted in a predominantly rural district of Pakistan that is already challenged in terms of resource availability. Hence, when generalizing the study findings, these may be interpreted in a similar context. Due to the researchers' experience and familiarity with the health system of the study district, the possibility of contamination of study results with researchers' own perceptions can not be completely eliminated, however, objectivity was ensured by note-taking during interviews and the use of interviews' audio recordings to ensure accuracy in data reporting.

CONCLUSION

We concluded that poor governance was perceived to lead to underutilization of existing resources for surveillance whereas lack of a policy framework on surveillance was considered to lead to a poor investment of resources in surveillance infrastructure. The absence of resources and inadequate human resource was identified by respondents as the key determinant of delayed and inadequate reporting leading to delayed detection of disease outbreaks. This was further aggravated by the absence of local laboratory capacity. The existing surveillance system was perceived as largely paper-based, slow, and comprised of hospital-based passive surveillance. The lack of private sector engagement in infectious disease surveillance was perceived as a significant gap.

RECOMMENDATIONS

The lack of directions from the provincial level regarding infectious disease surveillance necessitates the need for formulation of policy guidelines outlining not just the technical aspects of surveillance but also ensuring adequate resource planning and allocation to establish and sustain effective infectious disease surveillance at the district level. To ensure disease surveillance in the district, there is a need to i) address resource requirements

For peer review only - http://bmjopen.bmj.com/site/about/guidelines.xhtml

including adequate budget and human resource; ii) engage with the private health sector to capture maximum data of patients presenting to health facilities; iii) build active surveillance into the existing system by having designated human resource, iv) take advantage of the paperless system for data reporting to eliminate reporting delays and make real-time reporting system where data is instantly available after collection.

For peer review only

For peer review only - http://bmjopen.bmj.com/site/about/guidelines.xhtml

Funding

This work was supported by a grant from the World Health Organization grant number 1070479-0

Competing Interests

All authors declare that they have no competing interests

Authors contributions

IN, SS, RAS contributed to the plan and design of the study. IN developed the interview guide which RAS & RH reviewed and provided feedback. IN led data collection and performed data analysis. IN, SS, RAS & RH participated in the interpretation of the results. IN drafted the manuscript. SS, RAS, and RH contributed to revisions of the manuscript for intellectual content. All authors approved the final version of the manuscript.

Acknowledgement

We acknowledge all our participants for their cooperation during the conduct of this study

Checklist for the appropriate reporting statement

We used COnsolidated criteria for REporting Qualitative research (COREQ) checklist while writing the manuscript to ensure rigour in reporting the data for qualitative research

Patient consent for publication

Not applicable

Data availability statement

All the data collected as part of this research study are reported in the manuscript

Word count: 4744

List of abbreviations

TB – Tuberculosis

DHIS – District health information system

PDSRU – Provincial Disease Surveillance and Response Unit

CDC – Communicable Disease Control

PPP – Public-private partnership

VPDs – Vaccine preventable diseases

For peer review only

References

1. Ward JL, Azzopardi PS, Francis KL, et al. Global, regional, and national mortality among young people aged 10–24 years, 1950–2019: a systematic analysis for the Global Burden of Disease Study 2019. *Lancet* 2021;398(10311):1593-618.
2. Ledesma JR, Ma J, Vongpradith A, et al. Global, regional, and national sex differences in the global burden of tuberculosis by HIV status, 1990–2019: results from the Global Burden of Disease Study 2019. *Lancet Infect Dis* 2022;22(2):222-41.
3. World Health Organization. Communicable disease surveillance and response - Pakistan 2022 [cited 2022 July]. Available from: http://www.emro.who.int/pak/programmes/communicable-disease-a-surveillance-response.html accessed July 2022.
4. Ali M, Horikoshi YJPJMR. Situation analysis of health management information system in Pakistan. *Pakistan Journal Medical Research* 2002;41(2):64-9.
5. Zaidi SA, Bigdeli M, Langlois EV, et al. Health systems changes after decentralisation: progress, challenges and dynamics in Pakistan. *BMJ Glob Health* 2019;4(1):e001013.
6. Agency JIC. The District Health Information System (DHIS) Project for Evidence-Based Decision Making and Management 2009 [cited 2022 September]. Available from: https://www.jica.go.jp/pakistan/english/activities/activity02_03.html accessed September 2022.
7. Ahmad Z, Zahid HJJotPMA. Threat of a Measles outbreak amidst COVID 19 surge in Sindh, Pakistan-Letter to the editor. *J Pak Med Assoc* 2021;71(10):2488-88.
8. Siddiqui AR, Nathwani AA, Abidi SH, et al. Investigation of an extensive outbreak of HIV infection among children in Sindh, Pakistan: protocol for a matched case–control study. *BMJ Open* 2020;10(3):e036723.
9. World Health Organization. Inernational Health Regulations 2005 2022 [cited 2022 July]. Available from: http://apps.who.int/iris/bitstream/handle/10665/246107/9789241580496-eng.pdf;jsessionid=95E436B7F04EA4A8635945E4C684A14C?sequence=1 accessed July 2022.
10. Dehcheshmeh NF, Arab M, Foroushani AR, et al. Survey of communicable diseases surveillance system in hospitals of Iran: A Qualitative approach. *Glob J Health Sci* 2016;8(9):44.
11. Bilal W, Qamar K, Abbas S, et al. Infectious diseases surveillance in Pakistan: Challenges, efforts, and recommendations. 2022;78:103838.
12. Baig MA, Shaikh BTJAPJoPH. Disease surveillance system: a mandatory conduit for effective control of infectious diseases in Pakistan. 2012;24(4):586-94.
13. Abbasi IN, Siddiqi S. Overcoming the challenges of community-engaged emergency referrals in a rural district of Pakistan. *J Pak Med Assoc* 2021
14. Pakistan Bureau of Statistics. Thatta district 2017 [cited 2022 July]. Available from: https://www.pbs.gov.pk/census-2017-district-wise/results/091 accessed July 2022.
15. Pasha O, Saleem S, Ali S, et al. Maternal and 347 newborn outcomes in Pakistan compared to other low and middle income 348 countries in the Global Network's Maternal Newborn Health Registry: an 349 active, community-based, pregnancy surveillance mechanism. *Reprod Health* 2015;350:12.
16. National Institute of Population Studies Islamabad Pakistan. Pakistan Demographic and Health Survey 2017-18 [cited 2022 July]. Available from: https://dhsprogram.com/pubs/pdf/FR354/FR354.pdf accessed July 2022.
17. Shaikh B, Rabbani F, Safi N, et al. Contracting of primary health care services in Pakistan: is up-scaling a pragmatic thinking. 2010;60(5):387.
18. Makinde OA, Odimegwu COJPAMJ. A qualitative inquiry on the status and adequacy of legal instruments establishing infectious disease surveillance in Nigeria. *Pan Afr Med J* 2018;31(1)
19. Abuzerr S, Zinszer K, Assan AJSom. Implementation challenges of an integrated One Health surveillance system in humanitarian settings: A qualitative study in Palestine. *SAGE Open Med* 2021;9:20503121211043038.

For peer review only - http://bmjopen.bmj.com/site/about/guidelines.xhtml

543 20. Yan W, Zhou Y, Wei S, et al. The difficulties of early detection for infectious disease outbreak in China: a qualitative investigation. *Journal of Nanjing Medical University* 2008;22(1):66-70.
545 21. Angelo M, Ramalho WM, Gurgel H, et al. Dengue surveillance system in Brazil: A qualitative study in the federal district. *Int J Environ Res Public Health* 2020;17(6):2062.
547 22. Pilot E, Murthy G, Nittas VJGPH. Understanding India's urban dengue surveillance: A qualitative policy analysis of Hyderabad district. *Glob Public Health* 2020;15(11):1702-17.
549 23. Lu G, Liu Y, Beiersmann C, et al. Challenges in and lessons learned during the implementation of the 1-3-7 malaria surveillance and response strategy in China: a qualitative study. *Infect Dis Poverty* 2016;5(1):1-11.
552 24. Lagarde M, Palmer NJCDoSR. The impact of contracting out on health outcomes and use of health services in low and middle-income countries. *Cochrane Database Syst Rev* 2009(4)
554 25. Prajitha KC, Rahul A, Chintha S, et al. Strategies and challenges in Kerala's response to the initial phase of COVID-19 pandemic: a qualitative descriptive study. *BMJ Open* 2021;11(7):e051410.
557 26. Asadi H, Gouya M-M, Nabavi M, et al. The communicable diseases surveillance system in Iran: challenges and opportunities. *Arch Iran Med* 2019

For peer review only

For peer review only - http://bmjopen.bmj.com/site/about/guidelines.xhtml

COREQ (COnsolidated criteria for REporting Qualitative research) Checklist

A checklist of items that should be included in reports of qualitative research. You must report the page number in your manuscript where you consider each of the items listed in this checklist. If you have not included this information, either revise your manuscript accordingly before submitting or note N/A.

Topic	Item No.	Guide Questions/Description	Reported on Page No.
**Domain 1: Research team and reflexivity**			
Personal characteristics			
Interviewer/facilitator	1	Which author/s conducted the interview or focus group?	
Credentials	2	What were the researcher's credentials? E.g. PhD, MD	
Occupation	3	What was their occupation at the time of the study?	
Gender	4	Was the researcher male or female?	
Experience and training	5	What experience or training did the researcher have?	
Relationship with participants			
Relationship established	6	Was a relationship established prior to study commencement?	
Participant knowledge of the interviewer	7	What did the participants know about the researcher? e.g. personal goals, reasons for doing the research	
Interviewer characteristics	8	What characteristics were reported about the inter viewer/facilitator? e.g. Bias, assumptions, reasons and interests in the research topic	
**Domain 2: Study design**			
Theoretical framework			
Methodological orientation and Theory	9	What methodological orientation was stated to underpin the study? e.g. grounded theory, discourse analysis, ethnography, phenomenology, content analysis	
Participant selection			
Sampling	10	How were participants selected? e.g. purposive, convenience, consecutive, snowball	
Method of approach	11	How were participants approached? e.g. face-to-face, telephone, mail, email	
Sample size	12	How many participants were in the study?	
Non-participation	13	How many people refused to participate or dropped out? Reasons?	
Setting			
Setting of data collection	14	Where was the data collected? e.g. home, clinic, workplace	
Presence of non-participants	15	Was anyone else present besides the participants and researchers?	
Description of sample	16	What are the important characteristics of the sample? e.g. demographic data, date	
Data collection			
Interview guide	17	Were questions, prompts, guides provided by the authors? Was it pilot tested?	
Repeat interviews	18	Were repeat inter views carried out? If yes, how many?	
Audio/visual recording	19	Did the research use audio or visual recording to collect the data?	
Field notes	20	Were field notes made during and/or after the inter view or focus group?	
Duration	21	What was the duration of the inter views or focus group?	
Data saturation	22	Was data saturation discussed?	
Transcripts returned	23	Were transcripts returned to participants for comment and/or	

For peer review only

Topic	Item No.	Guide Questions/Description	Reported on Page No.
		correction?	
**Domain 3: analysis and findings**			
Data analysis			
Number of data coders	24	How many data coders coded the data?	
Description of the coding tree	25	Did authors provide a description of the coding tree?	
Derivation of themes	26	Were themes identified in advance or derived from the data?	
Software	27	What software, if applicable, was used to manage the data?	
Participant checking	28	Did participants provide feedback on the findings?	
Reporting			
Quotations presented	29	Were participant quotations presented to illustrate the themes/findings? Was each quotation identified? e.g. participant number	
Data and findings consistent	30	Was there consistency between the data presented and the findings?	
Clarity of major themes	31	Were major themes clearly presented in the findings?	
Clarity of minor themes	32	Is there a description of diverse cases or discussion of minor themes?	

Developed from: Tong A, Sainsbury P, Craig J. Consolidated criteria for reporting qualitative research (COREQ): a 32-item checklist for interviews and focus groups. *International Journal for Quality in Health Care*. 2007. Volume 19, Number 6: pp. 349 – 357

Once you have completed this checklist, please save a copy and upload it as part of your submission. DO NOT include this checklist as part of the main manuscript document. It must be uploaded as a separate file.

For peer review only

BMJ Open

Exploring stakeholders' experiences and perceptions regarding barriers to effective surveillance of communicable diseases in a rural district of Pakistan – a qualitative study

Journal:	*BMJ Open*
Manuscript ID	bmjopen-2022-067031.R2
Article Type:	Original research
Date Submitted by the Author:	25-Oct-2022
Complete List of Authors:	Naeem, Imran; Aga Khan University Medical College Pakistan, Community Health Sciences Department; Aga Khan University<br>Siddiqi, Sameen; Aga Khan University Medical College Pakistan, Community Health Sciences Department<br>Siddiqui, Amna Rehana; Aga Khan University, Community Health Sciences<br>Hasan, Rumina; The Aga Khan University Hospital Main Campus Karachi; London School of Hygiene and Tropical Medicine Faculty of Public Health and Policy
**Primary Subject Heading**:	Health services research
Secondary Subject Heading:	Health services research
Keywords:	International health services < HEALTH SERVICES ADMINISTRATION & MANAGEMENT, Organisation of health services < HEALTH SERVICES ADMINISTRATION & MANAGEMENT, Quality in health care < HEALTH SERVICES ADMINISTRATION & MANAGEMENT, Public health < INFECTIOUS DISEASES

SCHOLARONE™
Manuscripts

I, the Submitting Author has the right to grant and does grant on behalf of all authors of the Work (as defined in the below author licence), an exclusive licence and/or a non-exclusive licence for contributions from authors who are: i) UK Crown employees; ii) where BMJ has agreed a CC-BY licence shall apply, and/or iii) in accordance with the terms applicable for US Federal Government officers or employees acting as part of their official duties; on a worldwide, perpetual, irrevocable, royalty-free basis to BMJ Publishing Group Ltd ("BMJ") its licensees and where the relevant Journal is co-owned by BMJ to the co-owners of the Journal, to publish the Work in this journal and any other BMJ products and to exploit all rights, as set out in our licence.

The Submitting Author accepts and understands that any supply made under these terms is made by BMJ to the Submitting Author unless you are acting as an employee on behalf of your employer or a postgraduate student of an affiliated institution which is paying any applicable article publishing charge ("APC") for Open Access articles. Where the Submitting Author wishes to make the Work available on an Open Access basis (and intends to pay the relevant APC), the terms of reuse of such Open Access shall be governed by a Creative Commons licence – details of these licences and which Creative Commons licence will apply to this Work are set out in our licence referred to above.

Other than as permitted in any relevant BMJ Author's Self Archiving Policies, I confirm this Work has not been accepted for publication elsewhere, is not being considered for publication elsewhere and does not duplicate material already published. I confirm all authors consent to publication of this Work and authorise the granting of this licence.

For peer review only

TITLE:

Exploring stakeholders' experiences and perceptions regarding barriers to effective surveillance of communicable diseases in a rural district of Pakistan – a qualitative study

Imran Naeem[1*], Sameen Siddiqi[2], Amna Rehana Siddiqui[3], Rumina Hasan[4]

[1]Assistant Professor, Community Health Sciences, Aga Khan University Karachi Pakistan

[2]Professor & Chair, Community Health Sciences, Aga Khan University Karachi Pakistan

[3]Consultant, Community Health Sciences, Aga Khan University Karachi Pakistan

[4]Professor, Pathology & Laboratory Medicine, Aga Khan University Karachi Pakistan

[*]**Corresponding author**

Email: Imran.naeem@aku.edu

For peer review only

ABSTRACT

Objective: To explore the experiences and perceptions of health system stakeholders of a rural district of Sindh, Pakistan regarding the barriers to effective surveillance of communicable diseases.

Design: This qualitative descriptive exploratory design comprised in-depth interviews. Both inductive and deductive thematic analysis was applied to identify key themes from the data.

Settings: The study was conducted in public sector healthcare facilities and the district health office of the rural district of Thatta, in Sindh province, Pakistan.

Participants: Fifteen healthcare managers and healthcare providers working in the eight public sector primary and secondary healthcare facilities were interviewed using an open-ended in-depth interview guide.

Results: Key themes that emerged from the data were: poor governance and absence of surveillance policy framework; fragmentation in the health system leading to lack of uniform reporting; inadequate (human) resources that weakened the infrastructure for disease surveillance; hospital-based reporting of cases that led to a predominantly passive surveillance system; paper-based surveillance system as the key determinant of delayed reporting; non-utilization of surveillance data for decision making; absence of local laboratory capacity to complement the detection of disease outbreaks and lack of private sector integration in disease surveillance.

Conclusions: Poor governance and lack of policy framework were perceived to be responsible for weak surveillance infrastructure. Resource deficiencies including inadequate human resource, paper-based reporting and the absence of local laboratory capacity were considered to result in delayed, poor quality, and incomplete reporting. The lack of private sector engagement was identified as a major gap.

Keywords: surveillance, communicable diseases, health services research, qualitative, rural, Pakistan

For peer review only

For peer review only - http://bmjopen.bmj.com/site/about/guidelines.xhtml

Strengths and limitations of this study

- The study has explored experiences and perceptions regarding barriers to effective surveillance of communicable diseases in-depth by involving representatives from various levels of the healthcare system including from the public health sector and those working under public-private partnership
- The inclusion of both healthcare managers and healthcare providers in the study provided deeper insights into barriers at both the stewardship level and the operational level
- The study is amongst few in Pakistan to adopt a qualitative research approach for exploring barriers to infectious disease surveillance as perceived and experienced by health system stakeholders
- The study was conducted in a rural district of a developing country, hence study findings should be interpreted in a similar context

For peer review only

For peer review only - http://bmjopen.bmj.com/site/about/guidelines.xhtml

BACKGROUND

Infectious diseases continue to pose threat to the health of the public globally. In developing countries, infectious diseases form a significant portion of the disease burden including HIV/AIDS, tuberculosis (TB), malaria, respiratory infections, hepatitis B & C in adults and pneumonia and diarrhoea in children under five years of age [1 2]. In Pakistan, communicable diseases remain a major cause of public health concern with a significant contribution to morbidity and mortality. Conditions like overcrowding, low socio-economic status, poor hygiene and unsafe drinking water and poor awareness of health lead to an environment conducive to disease outbreaks. The Health System of the country is overwhelmed by issues of poor governance and lack of resources resulting in a surveillance system that is ill-equipped at detecting outbreaks of infectious disease [3]. Before the devolution of the health system, Pakistan had two main sources to collect data for health indicators namely: 1) health management information system: this was designed to collect data on selected health indicators from health facilities with established reporting lines from provincial to federal health ministry; 2) data from vertical programs such as national TB control program, malaria control program, HIV/AIDS control program amongst others that also reported to federal health ministry [4]. Following the health system devolution in 2010, administrative powers were devolved to provinces with the district becoming the autonomous unit for defining its health priorities and health planning [5]. In the new, albeit ill-prepared administrative setup district health information system (DHIS) was established to collect disease-related data from the health facility level [6]. In all provinces of the country including the Sindh province, DHIS is the only source of information on health indicators of the population based on service delivery data from public sector health facilities. However, the DHIS has remained underutilized for communicable disease surveillance. In the last five years, rural districts of Sindh have witnessed outbreaks of diseases such as measles [7] and HIV/AIDS [8] while the surveillance system was unable to predict these outbreaks. As per the international health regulations, member states of the World Health Organization have the obligation to develop and strengthen disease surveillance with the help of existing health system resources [9]. However, the emergence of recent outbreaks has indicated weaker surveillance of communicable diseases more so in rural areas of Pakistan. Moreover, only diseases with global priority (such as COVID-19 and Polio) have managed to get attention,

For peer review only - http://bmjopen.bmj.com/site/about/guidelines.xhtml

whereas surveillance for diseases of national priority has often struggled to compete for policy space and resources. The scientific literature on infectious disease surveillance in Pakistan is either quantitative in nature or mostly has discussed the implementation of models like the disease early warning system (DEWS) for surveillance [10-12]. However, there is a dearth of literature regarding the challenges that healthcare managers and providers face when implementing disease surveillance programs and perceptions and experiences of these healthcare professionals regarding barriers to effective surveillance of infectious diseases are less well studied in Pakistan. Such information will not only give an in-depth insight into the challenges faced in infectious disease surveillance but also inform the policy makers with recommendations for addressing those challenges. Hence, we conducted this study to explore the experiences and perceptions of health system stakeholders of a rural district of Sindh, Pakistan regarding the barriers to effective surveillance of communicable diseases.

METHODS

Study design and setting

This study used a qualitative descriptive exploratory design to explore the perceptions and experiences of district health system stakeholders regarding the barriers to an effective surveillance system for communicable diseases in the rural district of Thatta located in the province of Sindh, Pakistan. Thatta is situated approximately 100 kilometres from the provincial capital of Karachi. It is a predominantly rural district with an approximate population of 1 million [13 14]. Health indicators including maternal mortality ratio and neonatal mortality rate are amongst the worst in the country [15 16]. The situation of the healthcare system of Thatta is comparable to any other rural district of Pakistan with inadequate infrastructure and resources. There exist primary and secondary healthcare facilities in the district and there is also a private healthcare system in the district comprising general practitioner clinics and small hospitals. The study was conducted from February 15 to April 30, 2022, in eight public sector primary and secondary healthcare facilities in the district.

Study participants

For peer review only - http://bmjopen.bmj.com/site/about/guidelines.xhtml

We used purposive sampling to select study participants. Eligible participants were healthcare managers and healthcare providers working in the eight public sector primary and secondary healthcare facilities. Healthcare managers were those working at the district level and were responsible for the management of health services. Healthcare providers were the doctors responsible for the provision of clinical care at selected healthcare facilities. Participants included both males (n = 12) and females (n = 3). The age range of study participants was 29 – 57 years. The experience of study participants with the district health system ranged from 5 – 30 years.

We used the 'saturation principle' to determine the sample size for the study. Upon researchers' observation, with further interviews yielding no new or significant findings, data collection was stopped. This resulted in 15 in-depth interviews with healthcare managers and healthcare providers.

Data collection

The data were collected using an open-ended interview guide. The guide comprised questions and probes regarding perceptions and experiences about the current state of communicable disease surveillance and challenges in infrastructure and financing for surveillance, barriers in data reporting, timeliness of reporting, and quality of reported data. The guide was developed following a thorough literature review and the researchers' own experience and expertise on the subject. The guide was piloted before data collection. Based on the pretest results, researchers gained new insights and revised the interview guide by adding further questions and probes. The interviews were conducted by the first author (IN) having research experience in health systems, communicable diseases, and qualitative research. The first author moderated the interviews along with note-takers. The interviews were conducted in the local language (Sindhi) and were audiotaped. A debriefing session was held after each interview to reflect on the participants' responses. The interviews took place in health facilities at a time suitable for study participants with each interview lasting about 40 – 50 minutes.

Researchers' reflexivity

In qualitative research, research findings are liable to be influenced by researchers' interest and understanding of the topic. In this study, researchers were not the staff of the health

For peer review only - http://bmjopen.bmj.com/site/about/guidelines.xhtml

For peer review only

facilities where the study was conducted. However, due to the research team's four years of work experience in the district, they were not considered outsiders by the study respondents but rather someone familiar with the district health system and interested in exploring their views. Moreover, researchers used reflexive notes during data collection that fed into the interpretation of study findings to minimize researcher bias.

Researchers had knowledge of the health system and infectious diseases research which influenced the development of the interview guide, however, while pre-testing the guide researchers gained new knowledge which informed the revision of the interview guide.

Patient and public involvement statement

Patients or the public were not involved in the design, or conduct, or reporting, or dissemination plans of our research

DATA ANALYSIS

We used both inductive and deductive methods to perform data analysis. The analysis approach used a combination of interviews with study participants facilitated by field observations. Researchers' background knowledge of health systems and infectious disease research guided the process of data collection and analysis using the deductive method. However, during the analysis new themes emerged that were analysed using the inductive method. All audio tapes were transcribed from Sindhi to English and interview notes were written. Manual content analysis was performed where interview notes and notes from audio transcripts were read and re-read to identify patterns in data. Manual codes were assigned to identified patterns in data which were subsequently grouped and classified into main themes. All the authors and field team members read the themes, and discrepancies were discussed during the interpretation and analysis of data. The themes emerging from the analysis were shared and discussed with study participants for their comments.

RESULTS

Data analysis identified a number of core themes from in-depth interviews. Where appropriate, direct quotes from the interviews have been used to ensure rigour in the data reporting. To avoid identifying specific individuals, the participants have been allocated aliases. Table 1 summarizes the themes and codes that emerged from the data.

For peer review only - http://bmjopen.bmj.com/site/about/guidelines.xhtml

Table 1. Themes and codes emerging from data

Themes	Codes
The absence of a surveillance policy framework and poor governance leads to an ill-defined disease surveillance system	• Lack of provincial policy on infectious disease surveillance<br>• Lack of laws<br>• Lack of surveillance standards<br>• Lack of resource planning leading to dysfunctional digital information systems<br>• Lack of planning to ensure integrated surveillance of infectious diseases
Fragmentation in the healthcare system is a hindrance to a uniform reporting system	• Poor coordination between health system stakeholders<br>• Lack of integration between different levels of health facilities<br>• Lack of defined reporting lines for surveillance data<br>• Different organizations managing various levels of health facilities in the district
Inadequate resources translate to poor disease surveillance	• Inadequate provision of facilities and equipment<br>• Lack of dedicated human resource for surveillance<br>• Lack of financial support<br>• Lack of dedicated line item for surveillance in provincial/district budget
In the current system surveillance is predominantly passive	• Hospital-based surveillance<br>• Surveillance data collected from patients presenting to hospitals<br>• Lack of human resource to conduct surveillance in communities<br>• Low level of surveillance activities outside hospitals
Paper-based reporting is a key determinant of delayed disease reporting	• Surveillance data compiled on hard copies<br>• Hard copies are delivered from health facilities to the district health office

For peer review only - http://bmjopen.bmj.com/site/about/guidelines.xhtml

	• No dedicated human resource to transfer health facility reports to the district health office
Surveillance data is underutilized for evidence-based decision making	• Monthly report submission by health facilities to the district health office is mandatory<br>• No feedback was provided from the district health office to facilities on submitted reports<br>• Submitted reports are not reviewed for data errors<br>• No one from the district or provincial makes monitoring visits to check the fidelity of reported data
Lack of laboratory testing capacity takes a toll on disease surveillance	• There is no laboratory capable of conducting tests for diseases under surveillance<br>• Samples are sent to a regional laboratory for testing
The lack of integration of the private sector in disease surveillance is a major gap	• At the provincial or district level, no measures are taken to bring surveillance data from the private health sector into the mainstream<br>• The private health sector does not report surveillance data to the district health office or provincial health department except in case of COVID

The absence of a surveillance policy framework and poor governance leads to an ill-defined disease surveillance system

All study participants unanimously pointed out the lack of a comprehensive policy framework for disease surveillance. A respondent mentioned that despite that following devolution in the health system where the province has the autonomy of decision-making, no initiative regarding disease surveillance policy has been taken. As a result, there is a lack of clarity in reporting disease-related data, coordination between different stakeholders, and meaningful analysis of data and its use for taking action.

For peer review only - http://bmjopen.bmj.com/site/about/guidelines.xhtml

"National health policy emphasizes the importance of disease surveillance and having such a system in place; however, it is up to provinces to develop detailed guidelines for disease surveillance and ensure its implementation which is not happening! [Participant 14]."

Provincial disease surveillance and response unit

One of the study respondents mentioned that a digital information system named Provincial Disease Surveillance and Response Unit (PDSRU) was developed in 2016 with the support of donor money. The PDSRU had all the communicable diseases of importance listed and the system was expected to be linked up with secondary hospitals in the province for regular data collection. However, the PDSRU unit has remained dysfunctional and has not been utilized for the purpose it was built for.

"It is very unfortunate that we got the donor money to establish a surveillance system in the province, but we didn't plan for resources to make the system functional [participant 5]."

District health information system

One of the respondents said that the DHIS was developed and implemented in 2010 [6]. It was meant to collate service delivery data from primary and secondary health facilities with a monthly reporting frequency. The data is collected on paper at the health facility level and a hard copy of the monthly DHIS reporting form is handed over physically by each facility to the district health office. There, a computer operator enters the data into the digital portal of DHIS which then can be viewed on a dashboard.

In its current state, DHIS is the only functional health information system that has up-to-date disease information. But a study respondent pointed out that this information remains underutilized for disease surveillance as there is hardly any review and feedback on the reported data from higher levels (i.e., district health office or provincial health department). Moreover, since the data is collated and shared at the end of each month, its utility for detecting disease outbreaks is limited.

"DHIS data can be a very good source of passive surveillance, but we need to increase the reporting frequency from monthly to weekly and have regular reviews of the reported data [Participant 5]."

Communicable Disease Control unit

For peer review only - http://bmjopen.bmj.com/site/about/guidelines.xhtml

A study respondent mentioned that for infectious diseases, a Communicable Disease Control (CDC) unit was established at the provincial level in 2015. The intent was to integrate all the vertical (disease-specific) programs under one roof for planning, resource allocation and public health interventions. Under the CDC unit, blood-borne diseases like HIV/AIDS, hepatitis C and hepatitis B could be looked after single-handedly when addressing the mode of spread, designing interventions and surveillance. Unfortunately, at the district level, the CDC unit lacks infrastructure (e.g., dedicated building and office space, computers, and internet), dedicated human resources (district focal CDC person) and a plan ensuring how the integration of various diseases can take place.

"Having CDC presents an immediate opportunity for syndromic surveillance; we should not waste this opportunity [Participant 10]."

Fragmentation in the healthcare system is a hindrance to a uniform reporting system

Under the public-private partnership (PPP), the public sector healthcare system in Thatta has been contracted out to various private providers. Under PPP, these providers are responsible for providing health services on a day-to-day basis while the health department provides a budget[17].

One of the study respondents mentioned that in the Thatta district extensive contracting out of health services has been done and that each level of health facility (i.e., primary, and secondary) is managed by a different private partner. Distinct reporting lines and a lack of data-sharing mechanisms between private partners have given rise to fragmentation within the district health system.

"Primary level facilities are supposed to be linked with secondary level facilities for referrals and reporting of disease-related data, however, there are so many partners with each having its reporting line. This has negatively affected the reporting of disease surveillance [participant 10]."

A healthcare manager pointed out that in the Thatta district, surveillance for vaccine-preventable diseases (VPDs) is conducted by the district health office with the district surveillance coordinator as the focal point. Whereas notifiable diseases are reported by the focal persons of the disease-specific programs with a flow of information that is separate

For peer review only - http://bmjopen.bmj.com/site/about/guidelines.xhtml

from that of VPDs. This vertical nature of surveillance of different diseases is inefficient in terms of resource utilization as it creates parallel systems where surveillance activities are carried out in siloes. These inefficiencies in turn create a weaker surveillance system and put the population at higher risk for communicable disease outbreaks.

“Every disease-specific program such as that of tuberculosis, malaria etc reports its data in isolation, how can we have an integrated disease surveillance system in this situation? [Participant 7].”

Inadequate resources translate to poor disease surveillance

A healthcare manager highlighted the dearth of human resource for surveillance in the district. He mentioned that in the district health office, there is only one person (district surveillance coordinator) who is tasked to coordinate for surveillance of vaccine-preventable diseases (VPDs), data reporting and data entry into DHIS for all eight public sector health facilities of the district. The increased workload results in delayed data entry and delay in data transfer to district health stakeholders. Not only that he lacks a team of dedicated individuals for surveillance, but he also has no provision of transportation for reaching out to health facilities and communities for active surveillance. Most of the time, he has to rely on making telephone contact with each in charge of health facilities to get disease-related information.

All the study respondents mentioned that reporting for VPDs is mandatory and health facilities are required to send weekly reports (even for no cases called ‘zero reports’) of VPDs to the district surveillance coordinator. But due to a lack of resources e.g., transportation and dedicated surveillance staff, the reports from various health facilities in the district often get delayed.

“Expecting one person to lead disease surveillance in the district, in the absence of transportation and adequate human resource is too much to ask [Participant 2].”

A district healthcare manager pointed out that for notifiable diseases, the district focal persons of disease-specific programs have the responsibility of reporting data. The primary role of the focal person is program implementation which involves numerous tasks from planning and implementing to monitoring and reporting program activities. In absence of

For peer review only - http://bmjopen.bmj.com/site/about/guidelines.xhtml

adequate human resource, focal persons primarily rely on data reported as part of program implementation. This has limitations namely 1) this is a form of passive surveillance rather than active; 2) reporting of program data takes place at specific intervals whereas surveillance requires continuous monitoring of disease cases and prompt action. Many study respondents believed that these limitations lead to a disease surveillance system that struggles with the timely detection, and reporting of disease outbreaks.

In the current system, surveillance is predominantly passive

Study respondents pointed out that since the data collection is passive as it is collected largely from those patients that present to health facilities, hence it is a form of passive surveillance rather than active surveillance.

"We could be missing out on outbreaking at the community level since there is no one going and actively screening the community members [Participant 1]"

Respondents attributed the lack of community-based active surveillance to the lack of resources and mentioned that there is a need to increase resource allocation for strengthening active surveillance.

Paper-based reporting is a key determinant of delayed disease reporting

Several study respondents attributed the delayed reporting of surveillance data to paper-based reporting. The system for reporting health-related data from health facilities to the district health offices in Pakistan is paper-based for both VPDs and notifiable diseases. The health facility in charge fills out a zero-reporting form (for VPDs) or a case report (for a notifiable disease). A physical copy of this report is then sent to the district surveillance officer. This often gets delayed as someone must visit the health facility or district surveillance coordinator to deliver the physical copy of the report. To prevent delay, telephonic contact often plays a key role, however, it is not a feasible way to disseminate the information to all the relevant stakeholders.

"We should have a digital system with a dashboard which can show data in real-time. We will avoid delayed reporting forever [participant 2]."

For peer review only - http://bmjopen.bmj.com/site/about/guidelines.xhtml

Surveillance data is underutilized for evidence-based decision making

Most study respondents mentioned that data verification is essential to ensure its fidelity. To ensure data quality, monitoring and supervision of field staff and the facility healthcare staff sending data need strengthening. But all respondents agreed that in the existing system of disease surveillance, there is an emphasis on data collection only. No monitoring and supervision are happening from the district or provincial levels. Other than the circumstances where a suspected or confirmed case of disease under surveillance is reported, there is no action taken to verify the authenticity and quality of routinely reported data.

"Despite that, the data is regularly collected through DHIS and other channels, it is rarely reviewed or utilized for analysis or action [Participant 12]."

Lack of laboratory testing capacity takes a toll on disease surveillance

All the study respondents showed their concern regarding poor laboratory capacity for surveillance in the district. Among the eight public sector health facilities in the Thatta district, only two have clinical laboratories. However, none of these laboratories is equipped to conduct testing for any of the VPDs or notifiable diseases in the district. Biological samples collected from suspected patients are sent to a regional laboratory that is based in the capital city of the country (laboratory at National Institute of Health, Islamabad) situated at least 1000 kilometres from Thatta district.

A healthcare manager pointed out that the absence of a fully equipped laboratory nearby calls for measures to ensure proper storage and transportation of biological samples. This not only is resource-intensive but adds to the delays in the system staggered by issues of delayed reporting.

"Provincial health department should, at the least, take measures to build a laboratory in the province so that disease surveillance can be made a little more efficient [Participant 6]."

The lack of integration of the private sector in disease surveillance is a major gap

All the study respondents considered the absence of private-sector integration as an important gap. The private health sector is a major stakeholder in service delivery and caters to approximately 70% healthcare needs of the population. Unfortunately, however,

For peer review only

the service delivery data of the private health sector is not integrated into the DHIS of the public health sector. In fact, the government is still lagging in taking measures to regulate the private health sector in Sindh province.

One of the respondents said that integration of data from the private sector in the health information system is essential to ensure effective disease surveillance as currently, a significant chunk of the population's disease burden remains to be captured.

"Our disease surveillance will be at a loss from capturing the true disease burden unless it integrates data from the private health sector [Participant 6]."

DISCUSSION

Our study explored multifaceted barriers to effective surveillance of communicable diseases in a rural district of Pakistan. Our study showed that the lack of policy guidelines at the provincial level was fundamental to ineffective disease surveillance and poor data reporting. Poor stakeholders' coordination led to a lack of sharing of surveillance data hampering the surveillance efforts. Due to poor resource planning, the digital information systems i.e., PDSRU and DHIS built using donor resources were underutilized. Having the district surveillance coordinator as the sole person responsible for surveillance activities in the district, in absence of additional resources, was considered a major resource gap by study respondents. Most study respondents were concerned about the predominantly passive nature of existing district surveillance. Paper-based reporting together with inadequate human resource was considered an important cause of delayed reporting in surveillance. The lack of laboratory testing capacity in the district was another determinant for delayed reporting. There was an increasing emphasis on collecting data than using it for predicting outbreaks or taking measures to control these. The absence of inclusion of infectious disease surveillance data of private sector data in the district surveillance was identified as another major gap by study respondents.

Despite that a decade has elapsed since the health system in Pakistan was devolved, the pace of provinces taking charge of health planning and resource generation has remained rather sluggish. A study respondent mentioned that despite the increasing infectious disease outbreaks in Sindh and other provinces in recent years i.e., measles, HIV/AIDS and the global COVID pandemic, initiatives regarding comprehensive guidelines and laws for

For peer review only - http://bmjopen.bmj.com/site/about/guidelines.xhtml

disease surveillance are still lacking. Studies from Nigeria and Zambia show that the laws related to public health surveillance existed but were considered to be outdated and/or poorly implemented [18]. Studies from Iran and Palestine concluded that having laws and policies for disease surveillance enable governments to allocate funds for establishing surveillance programs and that health authorities should play a lead role in ensuring their implementation [10 19].

Having a dysfunctional PDSRU for reporting surveillance data is a classic example of relying on donor money instead of concentrating on building local capacity for a functional health information system. Many respondents termed this unfortunate and emphasized investing resources in reviving the PDSRU. In a study from China inadequacy of resources at the local level was identified as an important determinant of a functional digital information system for disease surveillance. The study reported that the top tier of government invests more in building digital information systems, but lower levels don't receive enough planned resources to ensure its implementation [20]. A functional digital information system has been found to speed up the reporting, improve data flow and ensure the availability of up-to-date data for the decision-makers. This eventually leads to early detection of and timely action against outbreaks [10].

Respondents in our study found the lack of adequate human resource for surveillance at the district level as concerning. Literature shows that for surveillance to be effective, adequate human resource is essential to undertake field-based surveillance and for efficient data reporting [21]. The resource deficiencies including field-based staff have been shown to undermine effective disease surveillance [19 22]. Moreover, putting the burden of surveillance activities on healthcare staff engaged in service delivery negatively affects their motivation, performance, and consequently the quality of reporting [10 20 23].

Despite several advantages of contracting out health services that the literature notes, the structural challenges largely remain unaddressed [24]. One such challenge is poor coordination between stakeholders. Many respondents pointed out that having multiple stakeholders in the same district had been detrimental to disease surveillance due to a lack of coordination and ambiguous reporting lines. The flow of data is independent of the level of health facility defeating the notion of integrated disease surveillance. Poor coordination between stakeholders [21 22] and between different levels of health facilities [10] have been reported to adversely affect the data reporting for disease surveillance.

For peer review only - http://bmjopen.bmj.com/site/about/guidelines.xhtml

Many study respondents pointed out the need to strengthen active surveillance in the district as in the present system, the surveillance was largely hospital-based where the data was being gathered from patients presenting to health facilities. Literature shows that the majority of people may opt not to show up for health seeking unless they develop serious symptoms [20]. This, in the event of a communicable disease, not only has the potential for the infection to spread but also leads to a delay in detecting an outbreak. Relying on passive surveillance only is often dependent on factors including patients' awareness, health-seeking behaviour and socioeconomic status and hence needs to be supplemented by some form of active surveillance [23].

Paper-based reporting in our study came out as an important barrier to timely reporting. The need for transitioning from slow, staff-reliant and paper-based reporting to the digital mode of reporting is increasingly recognized in literature [10 21]. A study from India has demonstrated improved disease notification and enhanced data reporting due to transitioning to digital media [25].

Except when a suspected or confirmed case of a disease is detected, data sent from health facilities is rarely reviewed at higher levels. Respondents in our study pointed out that there is more emphasis on data collection than its analysis and use. It is evident from the literature that providing regular feedback to facility staff on the data has been shown to act as a motivating factor and a performance boost [10 22].

Study respondents emphasized the need for having local laboratory capacity to ensure the timely detection of disease outbreaks. Studies in literature have demonstrated that in absence of a local laboratory, rapid diagnostics kits (RDTs) may facilitate confirming outbreak until laboratory test results become available [23] thus preventing delays that may incur in the transfer of biological samples [22].

The extent to which a health system can detect disease outbreaks is dependent on its capacity to capture patients' data. In Pakistan, the private health sector caters to most of the population's healthcare needs. However, it is largely unregulated in Sindh province and in Pakistan in general, leading to a lack of integration of its patient data with the public sector. Studies show that the poor private sector engagement in disease surveillance is an important issue hampering the surveillance efforts in many countries including India [22 25], Iran [26] and China [23].

For peer review only - http://bmjopen.bmj.com/site/about/guidelines.xhtml

LIMITATIONS OF THE STUDY

Our study was conducted in a predominantly rural district of Pakistan that is already challenged in terms of resource availability. Hence, when generalizing the study findings, these may be interpreted in a similar context. Due to the researchers' experience and familiarity with the health system of the study district, the possibility of contamination of study results with researchers' own perceptions cannot be completely eliminated, however, objectivity was ensured by note-taking during interviews and the use of interviews' audio recordings to ensure accuracy in data reporting.

RECOMMENDATIONS

The lack of directions from the provincial level regarding infectious disease surveillance necessitates the need for formulation of policy guidelines outlining not just the technical aspects of surveillance but also ensuring adequate resource planning and allocation to establish and sustain effective infectious disease surveillance at the district level. To ensure disease surveillance in the district, there is a need to i) address resource requirements including adequate budget and human resource; ii) engage with the private health sector to capture maximum data of patients presenting to health facilities; iii) build active surveillance into the existing system by having designated human resource, iv) take advantage of the paperless system for data reporting to eliminate reporting delays and make real-time reporting system where data is instantly available after collection.

CONCLUSION

We concluded that poor governance was perceived to lead to underutilization of existing resources for surveillance whereas lack of a policy framework on surveillance was considered to lead to a poor investment of resources in surveillance infrastructure. The absence of resources and inadequate human resource was identified by respondents as the key determinant of delayed and inadequate reporting leading to delayed detection of disease outbreaks. This was further aggravated by the absence of local laboratory capacity. The existing surveillance system was perceived as largely paper-based, slow, and comprised of hospital-based passive surveillance. The lack of private sector engagement in infectious disease surveillance was perceived as a significant gap.

For peer review only - http://bmjopen.bmj.com/site/about/guidelines.xhtml

Funding

This work was supported by a grant from the World Health Organization grant number 1070479-0

Competing Interests

All authors declare that they have no competing interests

Authors contributions

IN, SS, RAS contributed to the plan and design of the study. IN developed the interview guide which RAS & RH reviewed and provided feedback. IN led data collection and performed data analysis. IN, SS, RAS & RH participated in the interpretation of the results. IN drafted the manuscript. SS, RAS, and RH contributed to revisions of the manuscript for intellectual content. All authors approved the final version of the manuscript.

Acknowledgement

We acknowledge all our participants for their cooperation during the conduct of this study

Checklist for the appropriate reporting statement

We used COnsolidated criteria for REporting Qualitative research (COREQ) checklist while writing the manuscript to ensure rigour in reporting the data for qualitative research

Patient consent for publication

Not applicable

Data availability statement

All the data collected as part of this research study are reported in the manuscript

Word count: 5057

List of abbreviations

TB – Tuberculosis

DHIS – District health information system

PDSRU – Provincial Disease Surveillance and Response Unit

CDC – Communicable Disease Control

PPP – Public-private partnership

VPDs – Vaccine preventable diseases

Research Ethics Approval

This study involves human participants and was approved by the ethics review committee of the Aga Khan University Karachi Pakistan (reference # ERC # 2020-5777-15184). Participants gave informed consent to participate in the study before taking part.

For peer review only

For peer review only - http://bmjopen.bmj.com/site/about/guidelines.xhtml

References

1. Ward JL, Azzopardi PS, Francis KL, et al. Global, regional, and national mortality among young people aged 10–24 years, 1950–2019: a systematic analysis for the Global Burden of Disease Study 2019. *Lancet* 2021;398(10311):1593-618.
2. Ledesma JR, Ma J, Vongpradith A, et al. Global, regional, and national sex differences in the global burden of tuberculosis by HIV status, 1990–2019: results from the Global Burden of Disease Study 2019. *Lancet Infect Dis* 2022;22(2):222-41.
3. World Health Organization. Communicable disease surveillance and response - Pakistan 2022 [cited 2022 July]. Available from: http://www.emro.who.int/pak/programmes/communicable-disease-a-surveillance-response.html accessed July 2022.
4. Ali M, Horikoshi YJPJMR. Situation analysis of health management information system in Pakistan. *Pakistan Journal Medical Research* 2002;41(2):64-9.
5. Zaidi SA, Bigdeli M, Langlois EV, et al. Health systems changes after decentralisation: progress, challenges and dynamics in Pakistan. *BMJ Glob Health* 2019;4(1):e001013.
6. Agency JIC. The District Health Information System (DHIS) Project for Evidence-Based Decision Making and Management 2009 [cited 2022 September]. Available from: https://www.jica.go.jp/pakistan/english/activities/activity02_03.html accessed September 2022.
7. Ahmad Z, Zahid HJJotPMA. Threat of a Measles outbreak amidst COVID 19 surge in Sindh, Pakistan-Letter to the editor. *J Pak Med Assoc* 2021;71(10):2488-88.
8. Siddiqui AR, Nathwani AA, Abidi SH, et al. Investigation of an extensive outbreak of HIV infection among children in Sindh, Pakistan: protocol for a matched case–control study. *BMJ Open* 2020;10(3):e036723.
9. World Health Organization. Inernational Health Regulations 2005 2022 [cited 2022 July]. Available from: http://apps.who.int/iris/bitstream/handle/10665/246107/9789241580496-eng.pdf;jsessionid=95E436B7F04EA4A8635945E4C684A14C?sequence=1 accessed July 2022.
10. Dehcheshmeh NF, Arab M, Foroushani AR, et al. Survey of communicable diseases surveillance system in hospitals of Iran: A Qualitative approach. *Glob J Health Sci* 2016;8(9):44.
11. Bilal W, Qamar K, Abbas S, et al. Infectious diseases surveillance in Pakistan: Challenges, efforts, and recommendations. 2022;78:103838.
12. Baig MA, Shaikh BTJAPJoPH. Disease surveillance system: a mandatory conduit for effective control of infectious diseases in Pakistan. 2012;24(4):586-94.
13. Abbasi IN, Siddiqi S. Overcoming the challenges of community-engaged emergency referrals in a rural district of Pakistan. *J Pak Med Assoc* 2021
14. Pakistan Bureau of Statistics. Thatta district 2017 [cited 2022 July]. Available from: https://www.pbs.gov.pk/census-2017-district-wise/results/091 accessed July 2022.
15. Pasha O, Saleem S, Ali S, et al. Maternal and 347 newborn outcomes in Pakistan compared to other low and middle income 348 countries in the Global Network's Maternal Newborn Health Registry: an 349 active, community-based, pregnancy surveillance mechanism. *Reprod Health* 2015;350:12.
16. National Institute of Population Studies Islamabad Pakistan. Pakistan Demographic and Health Survey 2017-18 [cited 2022 July]. Available from: https://dhsprogram.com/pubs/pdf/FR354/FR354.pdf accessed July 2022.
17. Shaikh B, Rabbani F, Safi N, et al. Contracting of primary health care services in Pakistan: is up-scaling a pragmatic thinking. 2010;60(5):387.
18. Makinde OA, Odimegwu COJPAMJ. A qualitative inquiry on the status and adequacy of legal instruments establishing infectious disease surveillance in Nigeria. *Pan Afr Med J* 2018;31(1)
19. Abuzerr S, Zinszer K, Assan AJSom. Implementation challenges of an integrated One Health surveillance system in humanitarian settings: A qualitative study in Palestine. *SAGE Open Med* 2021;9:20503121211043038.

For peer review only - http://bmjopen.bmj.com/site/about/guidelines.xhtml

20. Yan W, Zhou Y, Wei S, et al. The difficulties of early detection for infectious disease outbreak in China: a qualitative investigation. *Journal of Nanjing Medical University* 2008;22(1):66-70.
21. Angelo M, Ramalho WM, Gurgel H, et al. Dengue surveillance system in Brazil: A qualitative study in the federal district. *Int J Environ Res Public Health* 2020;17(6):2062.
22. Pilot E, Murthy G, Nittas VJGPH. Understanding India's urban dengue surveillance: A qualitative policy analysis of Hyderabad district. *Glob Public Health* 2020;15(11):1702-17.
23. Lu G, Liu Y, Beiersmann C, et al. Challenges in and lessons learned during the implementation of the 1-3-7 malaria surveillance and response strategy in China: a qualitative study. *Infect Dis Poverty* 2016;5(1):1-11.
24. Lagarde M, Palmer NJCDoSR. The impact of contracting out on health outcomes and use of health services in low and middle-income countries. *Cochrane Database Syst Rev* 2009(4)
25. Prajitha KC, Rahul A, Chintha S, et al. Strategies and challenges in Kerala's response to the initial phase of COVID-19 pandemic: a qualitative descriptive study. *BMJ Open* 2021;11(7):e051410.
26. Asadi H, Gouya M-M, Nabavi M, et al. The communicable diseases surveillance system in Iran: challenges and opportunities. *Arch Iran Med* 2019

For peer review only

For peer review only - http://bmjopen.bmj.com/site/about/guidelines.xhtml

COREQ (COnsolidated criteria for REporting Qualitative research) Checklist

A checklist of items that should be included in reports of qualitative research. You must report the page number in your manuscript where you consider each of the items listed in this checklist. If you have not included this information, either revise your manuscript accordingly before submitting or note N/A.

Topic	Item No.	Guide Questions/Description	Reported on Page No.
**Domain 1: Research team and reflexivity**			
Personal characteristics			
Interviewer/facilitator	1	Which author/s conducted the interview or focus group?	
Credentials	2	What were the researcher's credentials? E.g. PhD, MD	
Occupation	3	What was their occupation at the time of the study?	
Gender	4	Was the researcher male or female?	
Experience and training	5	What experience or training did the researcher have?	
Relationship with participants			
Relationship established	6	Was a relationship established prior to study commencement?	
Participant knowledge of the interviewer	7	What did the participants know about the researcher? e.g. personal goals, reasons for doing the research	
Interviewer characteristics	8	What characteristics were reported about the inter viewer/facilitator? e.g. Bias, assumptions, reasons and interests in the research topic	
**Domain 2: Study design**			
Theoretical framework			
Methodological orientation and Theory	9	What methodological orientation was stated to underpin the study? e.g. grounded theory, discourse analysis, ethnography, phenomenology, content analysis	
Participant selection			
Sampling	10	How were participants selected? e.g. purposive, convenience, consecutive, snowball	
Method of approach	11	How were participants approached? e.g. face-to-face, telephone, mail, email	
Sample size	12	How many participants were in the study?	
Non-participation	13	How many people refused to participate or dropped out? Reasons?	
Setting			
Setting of data collection	14	Where was the data collected? e.g. home, clinic, workplace	
Presence of non-participants	15	Was anyone else present besides the participants and researchers?	
Description of sample	16	What are the important characteristics of the sample? e.g. demographic data, date	
Data collection			
Interview guide	17	Were questions, prompts, guides provided by the authors? Was it pilot tested?	
Repeat interviews	18	Were repeat inter views carried out? If yes, how many?	
Audio/visual recording	19	Did the research use audio or visual recording to collect the data?	
Field notes	20	Were field notes made during and/or after the inter view or focus group?	
Duration	21	What was the duration of the inter views or focus group?	
Data saturation	22	Was data saturation discussed?	
Transcripts returned	23	Were transcripts returned to participants for comment and/or	

For peer review only

Topic	Item No.	Guide Questions/Description	Reported on Page No.
		correction?	
**Domain 3: analysis and findings**			
Data analysis			
Number of data coders	24	How many data coders coded the data?	
Description of the coding tree	25	Did authors provide a description of the coding tree?	
Derivation of themes	26	Were themes identified in advance or derived from the data?	
Software	27	What software, if applicable, was used to manage the data?	
Participant checking	28	Did participants provide feedback on the findings?	
Reporting			
Quotations presented	29	Were participant quotations presented to illustrate the themes/findings? Was each quotation identified? e.g. participant number	
Data and findings consistent	30	Was there consistency between the data presented and the findings?	
Clarity of major themes	31	Were major themes clearly presented in the findings?	
Clarity of minor themes	32	Is there a description of diverse cases or discussion of minor themes?	

Developed from: Tong A, Sainsbury P, Craig J. Consolidated criteria for reporting qualitative research (COREQ): a 32-item checklist for interviews and focus groups. *International Journal for Quality in Health Care*. 2007. Volume 19, Number 6: pp. 349 – 357

Once you have completed this checklist, please save a copy and upload it as part of your submission. DO NOT include this checklist as part of the main manuscript document. It must be uploaded as a separate file.

For peer review only
